# Stereospecific Resistance to N2-Acyl Tetrahydro-β-carboline Antimalarials Is Mediated by a PfMDR1 Mutation That Confers Collateral Drug Sensitivity

**DOI:** 10.1021/acsinfecdis.4c01001

**Published:** 2025-01-14

**Authors:** Emily K. Bremers, Joshua H. Butler, Leticia S. Do Amaral, Emilio F. Merino, Hanan Almolhim, Bo Zhou, Rodrigo P. Baptista, Maxim Totrov, Paul R. Carlier, Maria Belen Cassera

**Affiliations:** † Department of Biochemistry and Molecular Biology, University of Georgia, Athens, Georgia 30602, United States; ‡ Center for Tropical and Emerging Global Diseases, University of Georgia, Athens, Georgia 30602, United States; § Department of Chemistry, Virginia Tech, Blacksburg, Virginia 24061, United States; ∥ Department of Pharmaceutical Sciences, University of Illinois Chicago, Chicago, Illinois 60612, United States; ⊥ Department of Medicine, Houston Methodist Research Institute, Houston, Texas 77030, United States; # MolSoft LLC, San Diego, California 92121, United States

**Keywords:** Plasmodium falciparum, malaria, tetrahydro-β-carbolines, drug resistance, PfMDR1, collateral drug sensitivity

## Abstract

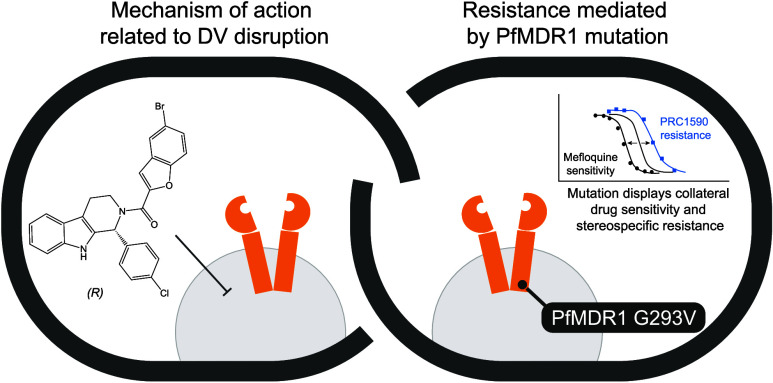

Half the world’s population is at risk of developing a malaria infection, which is caused by parasites of the genus *Plasmodium*. Currently, resistance has been identified to all clinically available antimalarials, highlighting an urgent need to develop novel compounds and better understand common mechanisms of resistance. We previously identified a novel tetrahydro-β-carboline compound, PRC1590, which potently kills the malaria parasite. To better understand its mechanism of action, we selected for and characterized resistance to PRC1590 in *Plasmodium falciparum*. Through *in vitro* selection of resistance to PRC1590, we have identified that a single-nucleotide polymorphism on the parasite’s multidrug resistance protein 1 (PfMDR1 G293V) mediates resistance to PRC1590. This mutation results in stereospecific resistance and sensitizes parasites to other antimalarials, such as mefloquine, quinine, and MMV019017. Intraerythrocytic asexual stage specificity assays have revealed that PRC1590 is most potent during the trophozoite stage when the parasite forms a single digestive vacuole (DV) and actively digests hemoglobin. Moreover, fluorescence microscopy revealed that PRC1590 disrupts the function of the DV, indicating a potential molecular target associated with this organelle. Our findings mark a significant step in understanding the mechanism of resistance and the mode of action of this emerging class of antimalarials. In addition, our results suggest a potential link between resistance mediated by PfMDR1 and PRC1590’s molecular target. This research underscores the pressing need for future research aimed at investigating the intricate relationship between a compound’s chemical scaffold, molecular target, and resistance mutations associated with PfMDR1.

Malaria represents a significant global health threat. Annually, more than 200 million individuals are infected with malaria, and over 600,000 deaths were reported in 2022 due to malaria infection.^1^ The causative agents of malaria are protozoan parasites from the *Plasmodium* genus, with *Plasmodium falciparum* responsible for the greatest number of deaths.^1^ Malaria is transmitted when an individual is bitten by an *Anopheles* mosquito harboring the *Plasmodium* parasite. Within 1–3 h of being bitten, malaria sporozoites travel to the liver where a cycle of asexual replication or schizogony begins. The schizogony process involves thousands of mitotic replications generating merozoites that are then released into the bloodstream, which allow them to invade red blood cells (RBCs) where the malaria parasite will rapidly replicate. During its asexual intraerythrocytic lifecycle, a merozoite progresses into a ring and then into the trophozoite stage, followed by schizogony. During schizogony, the parasite produces 18–36 daughter merozoites that are then released from the infected RBC, thus restarting its intraerythrocytic lifecycle.

Beginning in the ring stage, the parasite uptakes host RBC cytosol,^2^ forming a specialized lysosome-like organelle for digestion of host hemoglobin called the digestive vacuole (DV). The DV membrane is home to numerous transporters that facilitate the import and export of metabolites, lipids, and ions, which aid in the transport of hemoglobin products and maintain organelle ion-homeostasis.^3^ This organelle is an attractive drug target for antimalarials because the DV is specific to the malaria parasite, and dysregulation of heme detoxification or inhibition of DV transporters can lead to parasite death.^3,4^ Many antimalarials such as chloroquine target this organelle in the malaria parasite.^5−7^ Chemotherapies that successfully treat malaria infection, in combination with preventive measures such as vaccines and insecticides, are necessary to combat this global health threat. However, a major hurdle in controlling malaria in endemic regions is widespread antimalarial drug resistance. Unfortunately, drug resistance has been identified to all clinically approved antimalarials,^1^ highlighting an urgent need to develop novel antimalarial therapies and better understand common mechanisms of resistance in the malaria parasite.

One common mechanism of resistance in *P. falciparum* is mediated by point mutations and copy number variation of the multidrug resistance protein 1 (PfMDR1), which has been a marker of antimalarial drug resistance since the 1990s.^8−12^ PfMDR1 is an adenosine triphosphate (ATP)-binding cassette (ABC) transporter located in the membrane of the parasite’s DV that transports various substrates from the cytoplasm into this organelle. Interestingly, PfMDR1 mutations display collateral drug sensitivity, which occurs when the resistance mechanism for one compound contributes to the parasite’s sensitivity to another antimalarial drug.^12−15^ For example, amodiaquine resistance is associated with PfMDR1 mutations (i.e., N86Y and D1246Y)^16,17^ that result in increased sensitivity to mefloquine.^14^ Alternatively, mefloquine resistance is mediated by multiple copies of *pfmdr1*,^9,18^ which improves import of substrate into the DV.^19,20^ While PfMDR1 collateral drug sensitivity has been thoroughly documented in the literature,^12−14,17,21,22^ we do not fully understand what factors contribute to drug resistance mechanisms mediated by this transporter. It has previously been hypothesized that either the chemical scaffold^12,23,24^ or the localization of the molecular target of an antimalarial compound is related to the selection of PfMDR1 mutations.^3,6,15,19^ Understanding collateral drug sensitivity associated with PfMDR1 resistance can aid in the selection of better partner drugs that overcome drug resistance and help guide the synthesis of novel antimalarials.

Our previous work on pure enantiomers of N2-acyl tetrahydro-β-carbolines, a novel class of antimalarials, demonstrated their ability to potently kill *P. falciparum* malaria parasites.^25^ In the present work, we report the characterization of the mechanism of resistance and begin to assess the mechanism of action of a selected N2-acyl tetrahydro-β-carboline, named PRC1590. We have identified that resistance to PRC1590 is mediated by a single-nucleotide polymorphism (SNP) on the malaria PfMDR1 protein. We have characterized this mutation and explored the potential mechanism of action of PRC1590. Our findings indicate that both the mechanism of action and the chemical scaffold are important factors for collateral drug sensitivity associated with PfMDR1-mediated resistance.

## Results

### Selection of *P. falciparum* Parasites Resistant to PRC1590

We have previously reported our work on pure enantiomers of N2-acyl tetrahydro-β-carbolines and demonstrated that the (*R*)-configured compound, here named PRC1590, and its related tetrahydro-β-carbolines have potent *in vitro* antimalarial activity.^25^ We identified PRC1590 by searching a publicly available Novartis hit set of 5K antimalarial compounds^26,27^ for structures resembling the tetrahydro-β-carboline, MMV008138. We have previously studied MMV008138,^28−31^ a compound from the Malaria Box,^32^ that targets PfIspD enzyme in the methylerythritol phosphate (MEP) pathway.^29^ Our MMV008138-based similarity search returned the structure of GNF-Pf-5009, which is the racemic version of (*R*)-configured PRC1590. We then synthesized GNF-Pf-5009 and found that it produced a biphasic concentration-growth inhibition curve. The pure enantiomers were then prepared, and the (*R*)-enantiomer PRC1590 was found to display a normal monophasic dose-dependent growth inhibition curve while the (*S*)-enantiomer PRC1589 maintained pronounced biphasic behavior.^25^ Because of its monophasic and potent inhibition, we continued to pursue PRC1590. However, we found that PRC1590 and its derivatives do not target the MEP pathway as MMV008138.^25^

When possible, assessing the mechanism of resistance to novel antimalarials can aid in elucidating the compound’s molecular target.^33,34^ In order to evaluate the resistance mechanism of PRC1590 and gain deeper insights into its potential mode of action, we conducted single-step *in vitroP. falciparum* resistance selections in triplicate.^35^ Initially, we subjected the drug-sensitive *P. falciparum* strain 3D7 to PRC1590 at a concentration of 6 times the EC_50_ value for 14 days. Flasks containing an inoculum of 1 × 10^7^ parasites were used in this selection. In parallel, a flask treated with an equal volume of dimethyl sulfoxide (DMSO) was used as a reference control. After the drug pressure was removed, we continuously monitored the cultures for 60 days. However, we did not observe recrudescence in any of the flasks treated with PRC1590.

**Figure 1:**
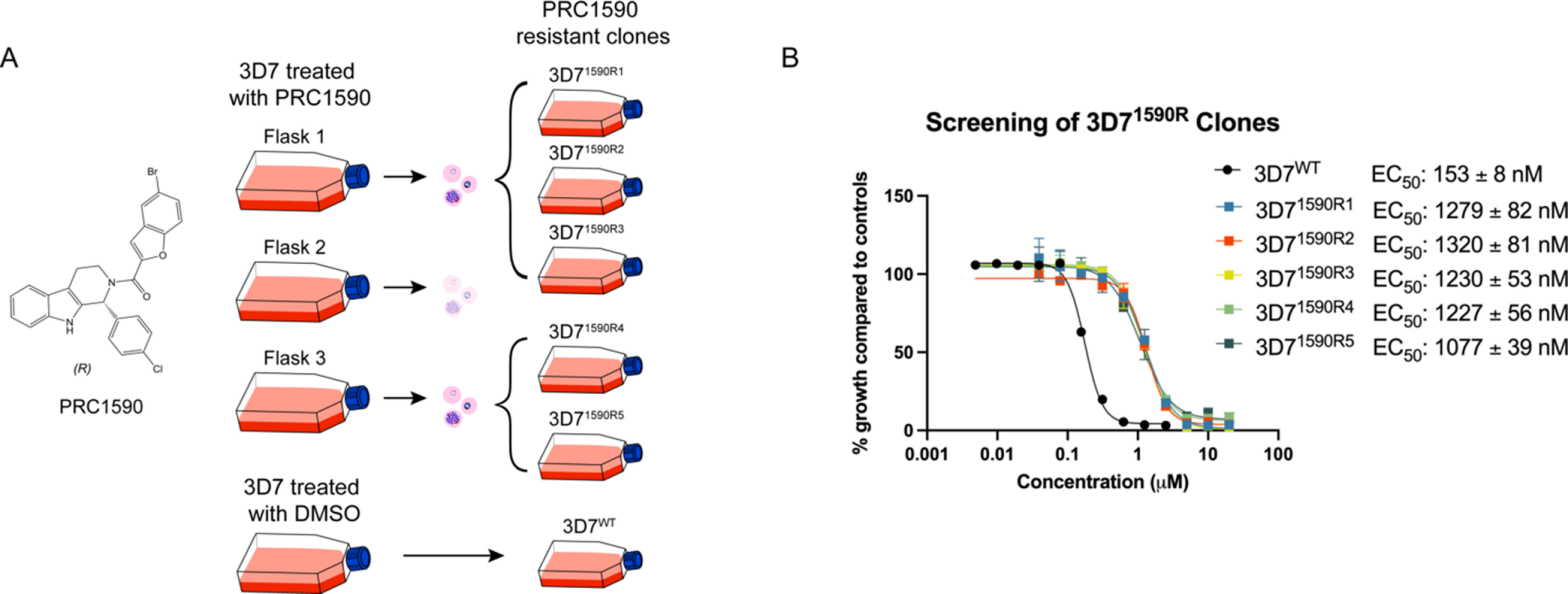
Selection of *P. falciparum* resistance to PRC1590. (A) Schematic of the single-step selection process used for selecting parasites resistant to PRC1590 and the structure of PRC1590. Three independent cultures containing an inoculum of 1 × 10^7^ of 3D7 parasites were subjected to 250 nM of PRC1590. A flask treated with the same volume of DMSO was maintained in parallel as a reference control. (B) Dose-dependent growth inhibition assays of 3D7^WT^ and each selected clone. A >5-fold increase in EC_50_ values between the resistant clones (3D7^1590R1–5^) and the wild-type line (3D7^WT^) was observed, indicating resistance to PRC1590. The *x*-axis is represented on a log scale. The reported values represent averages and SEM values of three independent assays.

We then conducted another single-step *in vitroP. falciparum* resistance selection for three independent cultures but at a concentration of 1.5 times the EC_50_ value and an inoculum of 1 × 10^7^ parasites of the 3D7 strain. Parasites treated with DMSO were also set as a reference control (Figure 1A). Drug pressure was maintained for 14 days and then removed, followed by continuous monitoring of the *P. falciparum* cultures. Through continuous monitoring of these cultures, we observed that parasites recrudesced in two of the three flasks 21 days following the start of selection. Five clones resistant to PRC1590 from these two independent cultures were isolated through limiting dilution, as shown in Figure 1 (3D7^1590R1–5^). To confirm resistance to PRC1590, we subjected 3D7^1590R1–5^ and their parental line exposed to DMSO (3D7^WT^) to dose-dependent growth inhibition assays with PRC1590. All selected clones had a >5-fold increase in their EC_50_ values compared to 3D7^WT^, indicating that the 3D7^1590R1–5^ clones were resistant to PRC1590 (Figure 1B). We monitored the stability of this resistance phenotype over four months of continuous culture without drug pressure and identified that the PRC1590 resistant clone 3D7^1590R1^ maintained a 5-fold increase in the EC_50_ value compared to 3D7^WT^ (Figure S1). In addition, we compared the growth phenotype between 3D7^WT^ and two resistant clones selected from flasks 1 and 3 (3D7^1590R1^ and 3D7^1590R4^). No visible differences in phenotypic development were observed between the 3D7^WT^ and the resistant lines (Figure S2).

### PRC1590 Resistant Line Displays Collateral Drug Sensitivity to Compounds with Known Mechanisms of Resistance Mediated by the Multidrug Resistance Protein 1 (PfMDR1)

**Figure 2:**
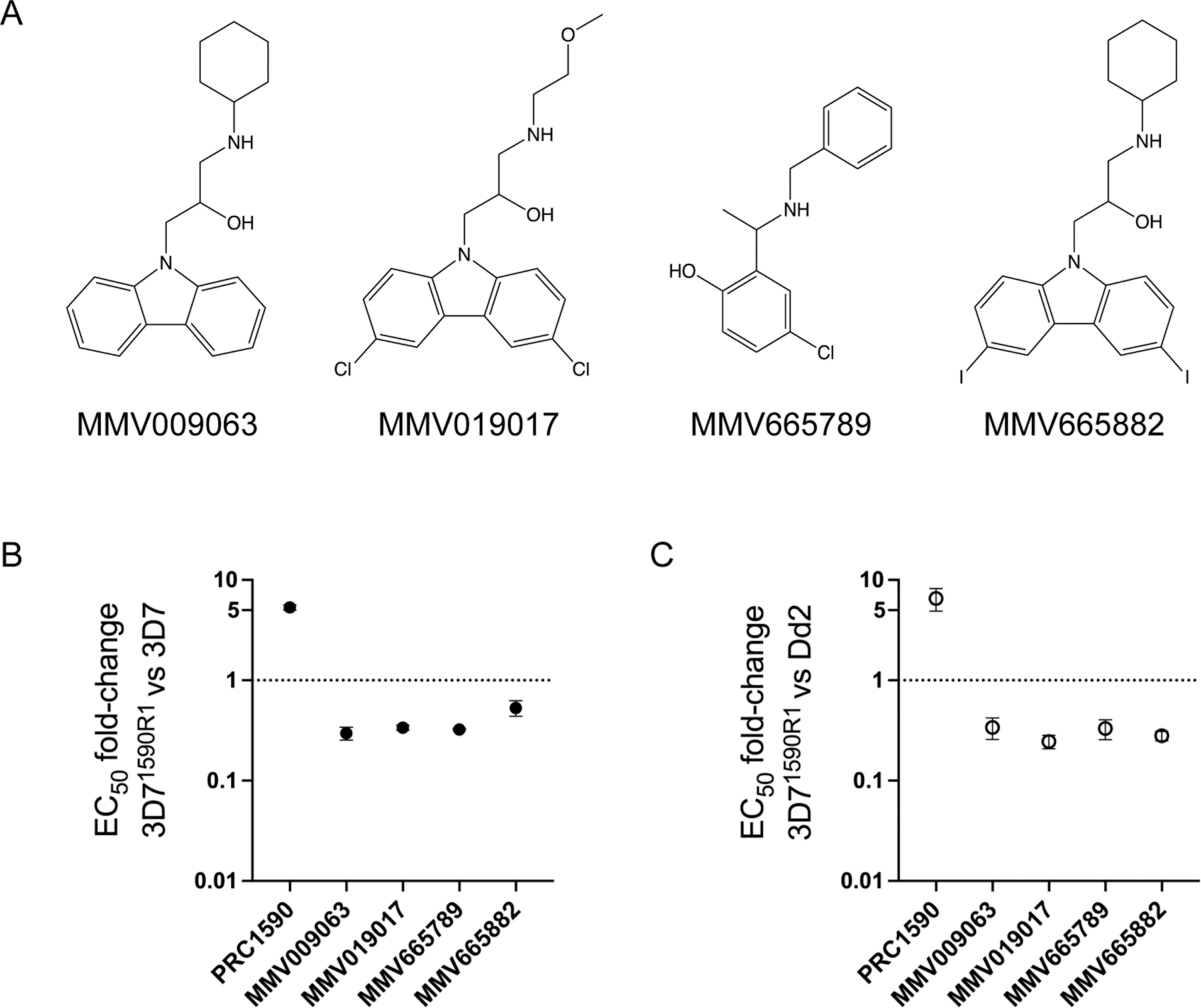
Cross-resistance screening of the 3D7^1590R1^ resistant line. (A) Chemical structures of the MMV compounds identified in the cross-resistance screening. MMV009063, MMV019017, and MMV665882 are quinoline methanol analogs. MMV665789 is a benzylamino-ethyl-phenol. (B) EC_50_ fold-changes between the 3D7^1590R1^ and 3D7^WT^ strains are shown for the selected MMV compounds. Fold-change values were calculated based on the EC_50_ values for 3D7^1590R1^ compared to 3D7^WT^. (C) EC_50_ fold-changes between 3D7^1590R1^ and Dd2 strains were calculated and show that 3D7^1590R1^ maintained increased susceptibility to the four tested MMV compounds when compared to the Dd2 strain. (B, C) The *y*-axes are shown on a log scale. The reported values represent averages and the SEM of two to three independent assays.

To begin characterizing the mechanism of resistance of the 3D7^1590R1–5^ clones, we assessed potential cross-resistance with antimalarials that have known mechanisms of resistance. Based on previous research that characterized the mechanism of resistance to compounds from the Malaria Box,^34,36,37^ we created a resistome library of 18 representative compounds with common mechanisms of resistance (Table S1). We then screened the 3D7^1590R1^ resistant clone in parallel with 3D7^WT^ against this resistome library. Interestingly, we identified that 3D7^1590R1^ was more susceptible to MMV009063, MMV019017, and MMV665789 compared to 3D7^WT^ (Figure 2A,B). Like mefloquine, these three compounds have known mechanisms of resistance associated with gene amplification of the *P. falciparum* multidrug resistance protein 1 (*pfmdr1*). We then expanded the cross-resistance screening to include MMV665882, a close analog of MMV009063 and MMV019017 in the Malaria Box, with a mechanism of resistance also mediated by gene amplification of *pfmdr1.*(34) We observed that 3D7^1590R1^ also showed increased sensitivity to MMV665882 when compared to 3D7^WT^ (Figure 2B).

**Figure 3:**
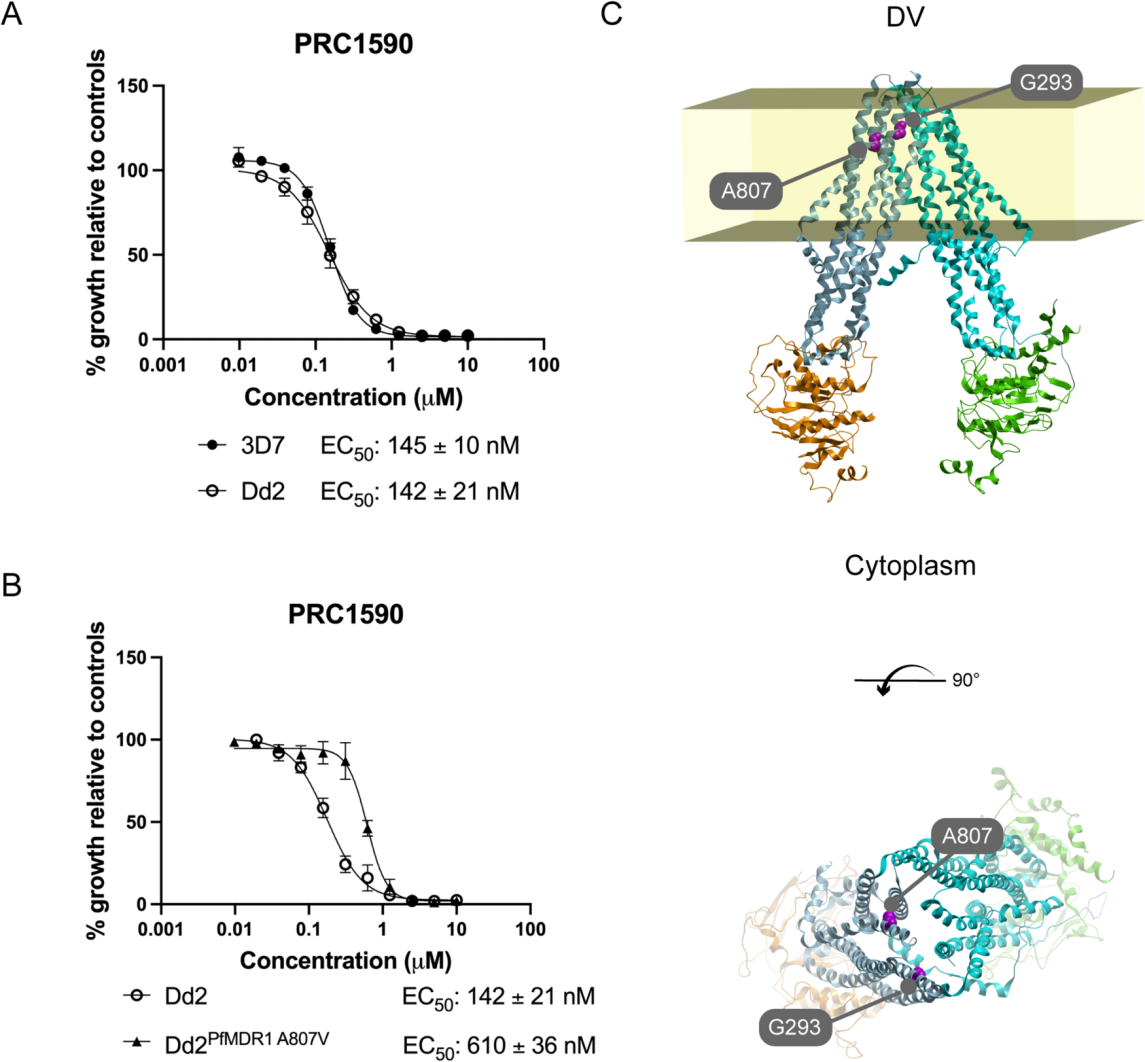
PfMDR1 A807V mutation displays cross-resistance to PRC1590. (A) PRC1590 dose-dependent growth inhibition assays in 3D7^WT^ and Dd2 strains. (B) Dose-dependent growth inhibition assays show an approximate 4-fold increase in the PRC1590 EC_50_ value in the Dd2^PfMDR1 A807V^ strain compared to the Dd2 wild-type. (A, B) The *x*-axes are shown on a log scale. The reported values represent averages and the SEM of three independent assays. (C) Homology model based on the apo crystal structure of PfMDR1 with highlighted resistance residues.^39^ PfMDR1 G293 and A807 are situated in transmembrane regions located near the DV.

To evaluate if copy number variation plays a role in mediating resistance to PRC1590, we tested PRC1590 and these four MMV compounds against the *P. falciparum* Dd2 strain, which has three copies of *pfmdr1.*(21,38) When compared to Dd2, we found that the 3D7^1590R1^ resistant clone displayed a 5-fold increase in the PRC1590 EC_50_ value and maintained increased susceptibility to the four MMV compounds (Figure 2C). This pattern was similar to the comparison of 3D7^1590R1^ to 3D7^WT^, which was unsurprising considering that Dd2 and 3D7 have similar EC_50_ values for PRC1590 (Figure 3A). These data suggest that copy number variation is likely not contributing to PRC1590 resistance. To further assess the potential role of PfMDR1 in the mechanism of resistance to PRC1590, we used a cell line of Dd2 that has multiple copies of *pfmdr1* and a PfMDR1 SNP (PfMDR1 A807V) present in one copy of the multidrug resistance gene. This is a resistant line that was selected after continuous exposure to the antimalarial candidate ACT-451840.^21^ The PfMDR1 A807V mutation is located in a transmembrane region close to the DV (Figure 3C). We observed that the Dd2 PfMDR1 A807V strain displayed a 4-fold increase in its PRC1590 EC_50_ value when compared to wild-type Dd2 (Figure 3B), similar to our 3D7^1590R1^ resistant line. These data suggest that polymorphism on the *pfmdr1* gene is likely mediating resistance to PRC1590.

### Resistance to PRC1590 Is Mediated by a SNP on *pfmdr1*

To confirm our hypothesis that resistance to PRC1590 is mediated by a SNP on the *pfmdr1* gene and not by gene amplification, we performed whole genome sequence analysis on the five selected PRC1590 resistant clones and their parental line exposed to DMSO (3D7^WT^). Whole genome sequence analysis revealed that all five clones derived from two independent culture selections, as shown in Figure 1 shared a single amino acid change on residue 293 of the PfMDR1 protein, resulting in a change from glycine to valine (Figure S2 and Table S3). The PfMDR1 G293V was the only mutation that matched our selection criteria of being absent in the parental 3D7^WT^ line but present in the coding region of a gene in all five resistant clones (Table S3). Herein, we refer to the drug-resistant clones as 3D7 PfMDR1 G293V. Additionally, we assessed gene amplification across all five clones, and no gene amplification of *pfmdr1* or other genes was detected. The PfMDR1 G293V mutation is located in a transmembrane region near the DV similar to PfMDR1 A807V, which has also displayed a 4-fold increase in its PRC1590 EC_50_ value when compared to wild-type Dd2 (Figure 3B,C). Overall, these findings support the results of our cross-resistance investigation described above, confirming our hypothesis that resistance to PRC1590 is mediated by an SNP on the *pfmdr1* gene rather than by gene amplification.

### PfMDR1 G293V Mutation Displays Stereospecific Resistance for N2-Acyl Tetrahydro-β-carbolines

**Figure 4:**
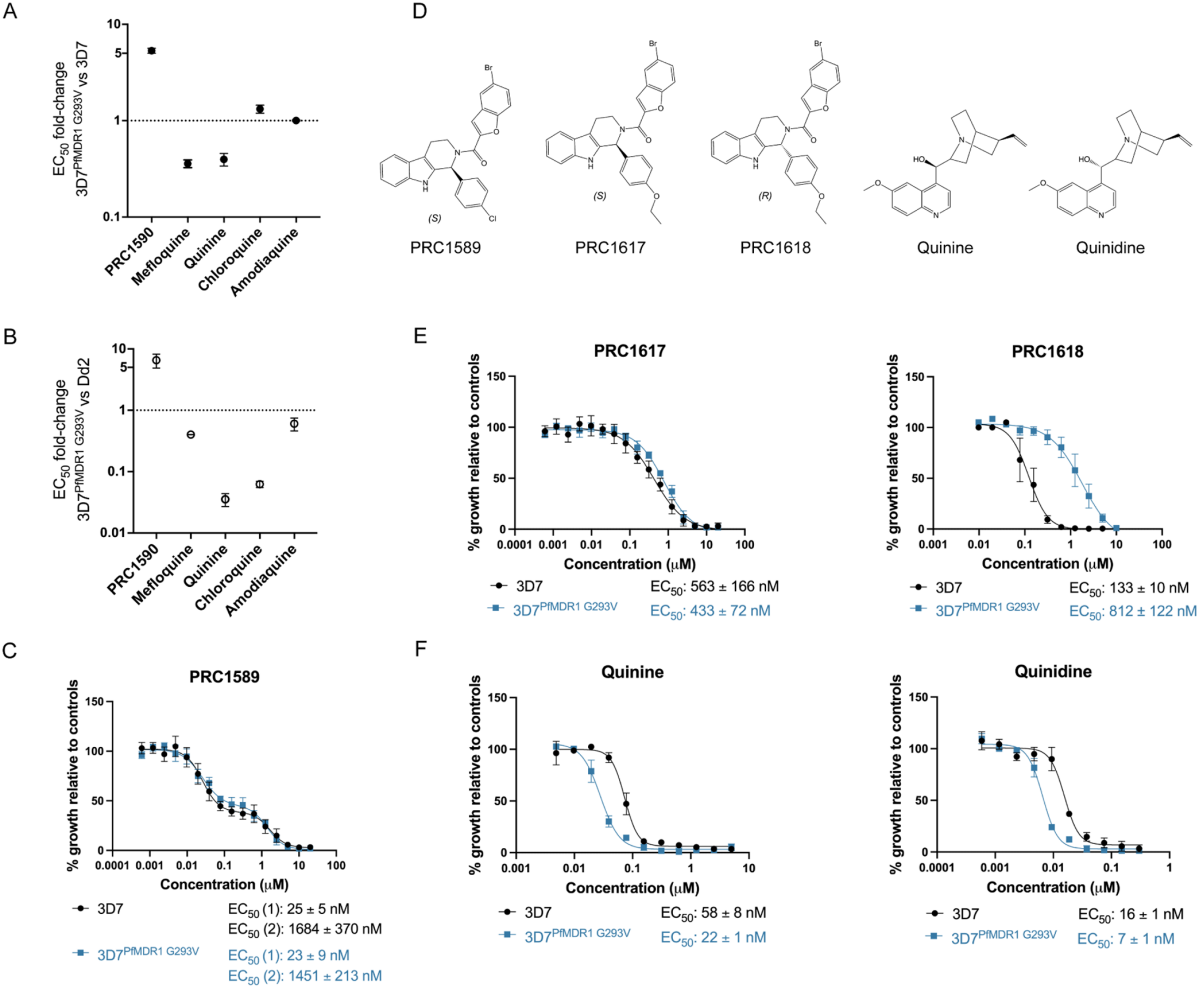
Expanded cross-resistance assessment of the PfMDR1 G293V strain (3D7^1590R1^). (A) Fold-change values were calculated based on the EC_50_ values of 3D7 PfMDR1 G293V compared to its parental line (3D7^WT^) for common antimalarials. 3D7 PfMDR1 G293V parasites are more sensitive to mefloquine and quinine, recapitulating collateral drug sensitivity associated with PfMDR1 mutations. The two 4-aminoquinolines, chloroquine and amodiaquine, showed no difference in the EC_50_ values when 3D7 PfMDR1 G293V was compared to 3D7^WT^. (B) EC_50_ fold-change between the PRC1590 resistant line and Dd2 strain shows that 3D7 PfMDR1 G293V is more sensitive to mefloquine, quinine, and chloroquine when compared to Dd2. (C) Dose-dependent growth inhibition assays show that the (*S*)-enantiomer of PRC1590 (PRC1589) remains equipotent in the PRC1590 resistant strain. (D) Structures of the tetrahydro-β-carbolines tested (PRC1589, PRC1617 and PRC1618), and structures of quinine and quinidine. (E) Dose-dependent growth inhibition assays indicate that the (*S*)-enantiomer of PRC1617 remains equipotent in the 3D7 PfMDR1 G293V strain, while the (*R*)-enantiomer PRC1618 displays around a 6-fold increase in EC_50_ value between 3D7 PfMDR1 G293V and 3D7^WT^. (F) Dose-dependent growth inhibition assays show that diastereomers quinine and quinidine display a similar decrease in the EC_50_ values. (A, B) The *y*-axes are shown on a log scale. (C, E, F) The *x*-axes are shown on a log scale. The reported values represent averages and the SEM of three independent assays.

To expand our cross-resistance screening, we subjected the 3D7 strain, two clones of 3D7 PfMDR1 G293V (3D7^1590R1^ and 3D7^1590R4^) derived from two independent flask selections, and the Dd2 strain to treatment with mefloquine, amodiaquine, chloroquine, and quinine. We continued to use the Dd2 strain in these cross-resistance assays to assess a wider spectrum of potential drug resistance and sensitivity, as PfMDR1-mediated resistance is pleiotropic and displays collateral drug sensitivity.^6,9,12^ Additionally, we selected these compounds because they have mechanisms of resistance mediated by or associated with PfMDR1 mutations. Resistance to mefloquine is mediated by gene amplification of *pfmdr1*,^14,18^ while amodiaquine resistance has been associated with PfMDR1 N86Y and D1246Y mutations.^16,17,22^ Chloroquine and quinine have resistance mechanisms mediated by PfCRT,^40,41^ another transporter present on the DV of the malaria parasite,^42^ and PfMDR1 (e.g., N86Y, D1246Y).^12,13,22^ Therefore, we assessed if the PfMDR1 G293V mutation displays cross-resistance with mefloquine, quinine, chloroquine, and amodiaquine by determining potential changes in the EC_50_ values relative to 3D7 and Dd2 strains. Through this cross-resistance analysis, we identified that 3D7 PfMDR1 G293V was more susceptible to mefloquine and quinine compared to 3D7 and Dd2 strains (Figure 4A,B and Table S2). An EC_50_ fold-change for chloroquine and amodiaquine was not observed in 3D7 PfMDR1 G293V when compared to 3D7^WT^. However, 3D7 PfMDR1 G293V was more sensitive than Dd2 to chloroquine, which was expected based on Dd2’s known resistance to chloroquine.^43^

Previously, it has been postulated that resistance mediated by PfMDR1 SNPs may be related to chemical scaffold.^23,24^ In other words, some scaffolds may be more susceptible than others to selection for mutations present in *pfmdr1*. Thus, we explored this hypothesis by extending the cross-resistance analysis to include the (*S*)-enantiomer of PRC1590, named PRC1589 (Figure 4C). As previously reported for Dd2 and 3D7 strains,^25^ the (*S*)-enantiomer PRC1589 continued to display a biphasic response in the 3D7 PfMDR1 G293V strain. Surprisingly, we observed no difference in the EC_50_ values of PRC1589 between 3D7^WT^ and 3D7 PfMDR1 G293V lines (Figure 4C), suggesting that the PfMDR1 G293V mutation results in stereospecific resistance. While the cause of the observed biphasic activity of PRC1589 remains to be determined, biphasic growth inhibition in *P. falciparum* has been attributed to direct inhibition of one target and downstream interaction with a second, functionally linked target.^44^

To further support the observation that the PfMDR1 G293V mutation displays stereospecific resistance, we tested a pair of analogs of PRC1589 and PRC1590, named PRC1617 and PRC1618 (Figure 4D). We similarly observed that the PRC1590 resistant strain showed resistance to the (*R*)-enantiomer PRC1618, while remaining sensitive to the (*S*)-enantiomer PRC1617 (Figure 4E). Based on these findings, we then tested quinidine, since it is the diastereomer of quinine, to assess if this stereospecificity pattern observed with the N2-acyl tetrahydro-β-carboline scaffold is maintained in a different antimalarial class. In contrast to N2-acyl tetrahydro-β-carbolines, we found that 3D7 PfMDR1 G293V maintained increased sensitivity to quinidine compared to 3D7 (Figure 4F). Despite testing a limited set of compounds, our cross-resistance analysis supports that PfMDR1 G293V mediates resistance to the (*R*)-enantiomer of the N2-acyl tetrahydro-β-carbolines. These data indicate that a chemical scaffold, even at the level of stereospecificity, can influence resistance mediated by PfMDR1. Further studies with diverse chemical scaffolds are necessary to establish the biochemical and structural basis of this selectivity.

### PRC1590 Acts Most Potently in the Trophozoite Stage of the Malaria Parasite

**Figure 5:**
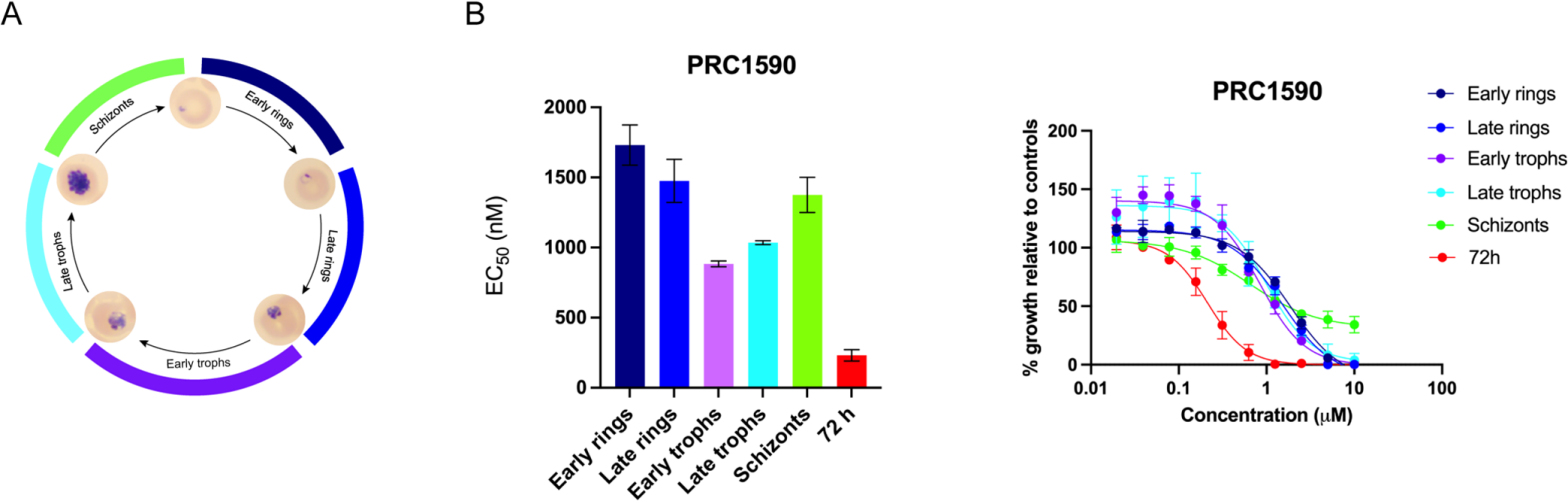
Stage specificity profiling. (A) Scheme of the intraerythrocytic life cycle of the malaria parasite showing the stages assessed for drug sensitivity profiling. Parasites were exposed to PRC1590 in a dose-dependent manner for 8 h starting in early rings, late rings, early trophozoites (trophs), late trophs, and schizonts. (B) PRC1590 acts most potently in the trophozoite stage of the malaria parasite. Stage specificity assays represent data from two biological replicates conducted in technical duplicate. The *x*-axis for the growth inhibition assay is shown on a log scale.

Previous research has identified that specific SNPs of PfMDR1 reduce the transport of antimalarials from the cytosol to the DV,^19,20^ presumably decreasing the concentration of drugs in the DV available to inhibit a specific target. Due to PRC1590 resistance being mediated by a DV transporter mutation, we predicted that PRC1590 would act most potently in the trophozoite stage since the DV coalesces and fully develops during this stage.^2^ To begin addressing this hypothesis, we used the stage specificity assay previously described by Murithi and colleagues,^44^ which aids in identifying potential modes of action for new antimalarial based on drug susceptibility profiles of previously characterized antimalarials. For these assays, we used chloroquine as a control because its mechanism of action is related to inhibition of heme degradation in the DV,^45−48^ and the stage-specific susceptibility profile of chloroquine has been previously characterized.^44^ As shown in Figure 5, we identified that the potency of PRC1590 increased in early and late trophozoite stages, while chloroquine was detected to be most potent as early as the ring stage (Figure S4).^44^ Additionally, no noticeable morphological changes were observed in the ring and trophozoite stages when 3D7 strain parasites were treated with PRC1590. However, PRC1590-treated parasites did not progress to schizont stage, but instead condensed late-stage trophozoites were observed, while treatment with chloroquine resulted in an enlarged DV (Figure S4B).^6^ Therefore, we hypothesize that PRC1590 has a mechanism of action associated with *P. falciparum*’s DV that differs from chloroquine.

### PfMDR1 G293V Mutation Does Not Impact Import of Fluo-4

**Figure 6:**
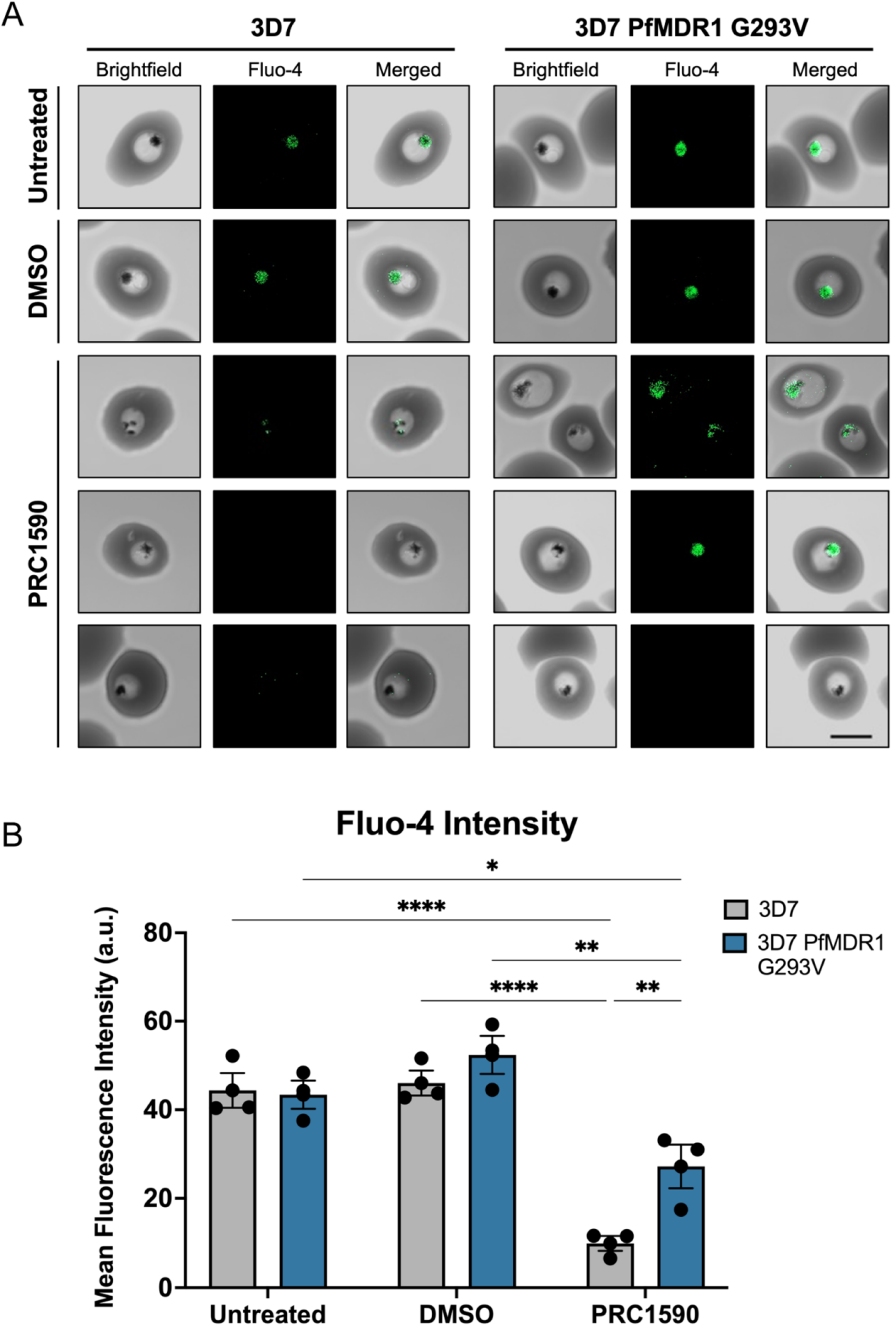
Assessment of PfMDR1 import and DV disruption using the Fluo-4 AM probe. (A) Representative images of Fluo-4 AM assays performed in 3D7 and 3D7 PfMDR1 G293V parasites. For each condition, *n* = 30 images were taken across three biological replicates. Scale bar = 5 μm. Additional images for each condition are available in the Supporting Information (Figures S5–S11). (B) Quantification of fluorescence intensity of Fluo-4 for the 3D7 strain and 3D7 PfMDR1 G293V line untreated, treated with DMSO, and treated with PRC1590. The reported values represent averages and the SEM from three biological replicates. Two-way analysis of variance (ANOVA) and Šídák’s tests were performed to compare treatment conditions and only statistically significant comparisons are shown. (****) *p* < 0.0001, (**) *p* < 0.001, (*) *p* < 0.05.

Previous studies have identified that specific SNPs of PfMDR1 can reduce the transport of Fluo-4^19,20^ into the DV. The Fluo-4 probe is a cell-permeant dye used to measure intracellular calcium concentrations. In *Plasmodium*, Fluo-4 is predominantly visible in the DV despite this organelle being a secondary store of calcium due to the pH dependence of this dye.^49^ In order to assess the impact PfMDR1 G293V has on import, we conducted Fluo-4 import assays in the resistant cell line and the wild-type 3D7 line. As expected, the wild-type 3D7 strain displayed Fluo-4 localization within the DV of the parasite (Figure 6A).^50^ We also observed that the 3D7 PfMDR1 G293V strain displayed similar fluorescence intensity as its wild-type parental line (Figure 6), indicating that the PfMDR1 G293V mutation does not impact the import of Fluo-4 into the DV.

### PRC1590 Treatment Disrupts the Function of the DV

While we did not identify differences in import between the wild-type 3D7 and the PRC1590 resistant line, we wanted to assess how treatment with PRC1590 may affect the DV function of these two strains. Previous studies have used Fluo-4 AM to assess whether compounds block PfMDR1 import^51^ or affect DV membrane integrity that leads to programmed cell death (PCD).^52,53^ For example, treatment with chloroquine and other lysomotrophic compounds affected DV membrane integrity, thus resulting in PCD.^54^ We observed that the 3D7 wild-type strain had reduced Fluo-4 fluorescence intensity and, similar to previously reported, parasites would occasionally display a diffuse Fluo-4 pattern when treated with chloroquine (Figure S7).^52,55^ We also observed that parasites treated with PRC1590 had a significant reduction in Fluo-4 fluorescence intensity, suggesting that PRC1590 may disrupt the DV structure or function for both the 3D7 wild-type and the PRC1590 resistant lines (Figure 6B). While treatment with PRC1590 significantly reduced mean fluorescence intensity for 3D7 and 3D7 PfMDR1 G293V compared to the untreated and DMSO conditions, we observed that the resistant line showed significantly greater mean fluorescence intensity compared to that of its parental line when treated with PRC1590 (Figure 6B). These results suggest that 3D7 PfMDR1 G293V parasites better tolerate PRC1590, potentially by mitigating the disruption of DV structure or function. Additionally, these data further support the hypothesis that PRC1590’s mode of action is related to this organelle in the malaria parasite.

## Discussion

Antimalarial drug resistance is prevalent and widespread, highlighting a critical need to develop novel therapeutics against malaria. Both β-carbolines and tetrahydro-β-carbolines have shown therapeutic potential as antimalarials.^28,29,31,56−58^ Our previous work on pure enantiomers of the N2-acyl tetrahydro-β-carboline GNF-Pf-5009, named here PRC1589 and PRC1590, led us to pursue PRC1590 as a potential antimalarial candidate against *P. falciparum*.^25^ In order to understand the liabilities of this compound, we aimed to characterize the mechanism of resistance and action of PRC1590. In this work, we identified that the mechanism of resistance to PRC1590 is mediated by the G293V SNP of the *P. falciparum* multidrug resistance protein 1 (PfMDR1), a transporter located on the membrane of the DV. PfMDR1 mutations are a common form of resistance, often acting as molecular markers of antimalarial drug resistance.^8−12,59^ The PfMDR1 G293V mutation has not been documented in the field based on a search of the open-source database, MalariaGEN.^60^ However, while this paper was under revision, a new study was published reporting the *in vitro* selection of PfMDR1 G293V mutation by the antiviral lopinavir, an HIV protease inhibitor that has been shown to have activity against *P. falciparum*.^61^ To better understand how PRC1590 works as an antimalarial, we characterized its resistance mechanism mediated by PfMDR1 G293V and explored its mode of action with fluorescence microscopy.

Through cross-resistance screenings, we found that PfMDR1 G293V sensitizes parasites to mefloquine, quinine, and the tested quinoline methanol analogs when compared to its wild-type parent line (Figures 2B and 4A). Mutations of PfMDR1 commonly display collateral drug sensitivity, which occurs when a mutation that mediates resistance to one drug contributes to the parasite’s sensitivity to another antimalarial drug.^12−15^ Mefloquine and the quinoline methanol analogs identified in our resistome assays often select for multiple copies of *pfmdr1* to mediate resistance,^18,34^ which is associated with improved import of substrates into the DV.^20,50^ It has previously been hypothesized that multiple copies of *pfmdr1* can reduce the potency of compounds with targets outside the DV by sequestering drugs in this organelle, away from their target.^3,14,19,24^ Despite having limited knowledge about the mechanisms of action for many antimalarials, the targets for MMV019017 and mefloquine are predicted to be outside the DV. Compound MMV019017 is thought to inhibit PfATP4, an ATPase transporter located on the plasma membrane of the malaria parasite.^44,62^ On the other hand, the mechanism of action of mefloquine is still debated, and evidence suggests that mefloquine either inhibits hemoglobin import in the cytoplasm^15,63^ or binds directly to PfMDR1.^39^ These findings suggest that PfMDR1 G293V can sensitize parasites to compounds with targets outside of the DV and/or antimalarials with mechanisms of resistance that select for multiple copies of *pfmdr1*. Both possibilities align with previous hypotheses that collateral drug sensitivity associated with PfMDR1 resistance is related to the molecular targets of antimalarials.^3,6,15^

To better understand the collateral drug sensitivity associated with PRC1590 resistance, we performed cross-resistance screenings with other cell lines containing PfMDR1 mutations. We used the Dd2 strain, which contains multiple copies of *pfmdr1*, and Dd2 PfMDR1 A807V, a line possessing multiple copies of *pfmdr1* and a SNP that mediates resistance to ACT-451840, a preclinical candidate antimalarial.^21^ ACT-451840 resistance can be mediated by multiple SNPs including PfMDR1 Y290F, a residue near the G293V mutation, and PfMDR1 A807V.^21^ We identified that Dd2 PfMDR1 A807V confers resistance to PRC1590 (Figure 3B). Additionally, similar to PfMDR1 G293V, the PfMDR1 A807V and PfMDR1 Y290F mutations sensitize parasites to mefloquine and quinine.^21^ These findings suggest that nearby PfMDR1 SNPs may display similar collateral drug sensitivity profiles.

To further characterize the PfMDR1 G293V mutation, we conducted PfMDR1 import assays. Interestingly, PfMDR1 G293V may not impact the import of PRC1590 into the DV like other reported PfMDR1 SNPs.^15,19,20,50^ We surprisingly did not observe differences in Fluo-4 fluorescence intensity in the DV between the 3D7 and 3D7 PfMDR1 G293V lines (Figure 6). Previous research has identified that specific SNPs of PfMDR1 (e.g., Y184F, N1042D, D1246Y) result in reduced import of Fluo-4 into the DV.^19,20,50^ Our data indicate that the PfMDR1 G293V mutation does not impact the import of Fluo-4 (Figure 6B), suggesting that this mechanism of resistance may not be related to PRC1590 import into the DV.

To begin to explore the mechanism of action of PRC1590, we conducted stage specificity assays. We identified that peak activity for PRC1590 was observed in the trophozoite stage of the malaria parasite (Figure 5), when the DV is fully formed.^2,3^ Additionally, we observed that treatment with PRC1590 disrupts the DV structure or function of the malaria parasite, potentially by affecting DV membrane integrity,^52^ blocking PfMDR1 import,^51^ or disrupting calcium dynamics in the DV^55^ (Figures 6 and S5–S11). These results resemble previously studied compounds that selected for similar PfMDR1 SNPs to PfMDR1 G293V and shown to interact with the DV membrane itself or transporters located on the DV membrane.^64,65^ For example, ACT-451840 is predicted to target the membrane of the DV,^63^ and an analog of ACT-451840 has been experimentally shown to bind to PfMDR1.^64^ Additionally, recent work characterizing antiplasmodial peptaibols identified that PfMDR1 G293C confers low level of resistance to HZ NPDG-I, a potent peptaibol against malaria.^65^ Further characterization of HZ NPDG-I identified that this compound disrupts the DV membrane of the malaria parasite by bending or forming a barrel-stave channel in the DV membrane.^65^ Taken together, our findings and previous work suggest that PfMDR1 G293V and related SNPs may mediate resistance to compounds targeting DV function, which further suggests a link between the mode of action and the mechanism of resistance. However, additional work is necessary to identify the precise mechanism of action of PRC1590.

While our data suggest that mechanism of resistance, collateral drug sensitivity, and mode of action are linked, we also observed that chemical scaffold was an important factor in the resistance mediated by PfMDR1 G293V. Through cross-resistance screenings, we found that the (*R*)-enantiomer of two N2-acyl tetrahydro-β-carbolines (PRC1590 and PRC1618) were 5- to 6-fold less potent in the 3D7 PfMDR1 G293V line compared to the wild-type 3D7 strain (Figure 4A,E). In contrast, the (*S*)-enantiomers PRC1589 and PRC1617 remained equally potent in the wild-type 3D7 strain and 3D7 PfMDR1 G293V line (Figure 4C,E), indicating that chemical structure plays a role in the mechanism of resistance mediated by this PfMDR1 mutation.

Additionally, we identified that the 4-aminoquinolines, chloroquine and amodiaquine, remained equally potent in the parental 3D7 strain and 3D7 PfMDR1 G293V line. We initially hypothesized that the PRC1590 resistant strain would show reduced sensitivity to these 4-aminoquinolines because resistance to these compounds is associated with PfMDR1 mutations (i.e., N86Y & D1246Y)^12,16,17^ and they have predicted molecular targets in the DV.^5,45,66^ However, some PfMDR1 SNPs can compensate for resistance mechanisms conferred by other genes or improve parasite’s overall fitness.^13,67−69^ Additionally, chloroquine and amodiaquine act as weak bases and likely passively diffuse through the DV membrane, bypassing active transport through PfMDR1.^70,71^ Thus, PfMDR1 G293V may not greatly affect unprotonated 4-aminoquinolines from reaching their targets.

We also identified that 3D7 PfMDR1 G293V parasites were more sensitive to quinine, despite quinine localizing to the DV and having predicted targets in the DV.^72,73^ These findings suggest that PfMDR1 G293V may affect the import of quinine into the DV, as other PfMDR1 mutations (i.e., Y184F and N1042D) have experimentally improved transport of quinine.^15^ Our data suggest that various mutations of PfMDR1 differentially affect the transport of specific chemical scaffolds,^15,24^ supporting the hypothesis that chemical structure also influences drug resistance and sensitivity for parasites harboring PfMDR1 mutations.

In summary, the work described here supports that PRC1590 has a molecular target associated with the DV of the malaria parasite, which remains to be identified, and further supports a link between PfMDR1 SNPs and potential molecular targets related to the DV of the malaria parasite.^72^ In addition, our study suggests that the chemical scaffold, including stereospecificity, is related to drug resistance and collateral drug sensitivity associated with the PfMDR1 G293V mutation. Lastly, our findings may inform future synthesis efforts for scaffolds that target processes in the DV which is a validated and valuable malaria-specific drug target.

## Materials and Methods

### *P. falciparum* Growth Inhibition

Dose-dependent growth inhibition with the reported compounds was evaluated using a 10-point dilution series and *in vitro* SYBR Green I assay as a readout. Briefly, ring-stage parasites (1% starting parasitemia and 1% hematocrit) were treated continuously with each compound in 96-well half-area dark plates for 72 h at 37 °C in reduced oxygen conditions (5% CO_2_, 5% O_2_, and 90% N_2_). After completing the 72 h incubation, growth was assessed by the SYBR Green I assay, as previously described.^74^ SYBR Green I was excited at 485 nm, and its emission was measured at 535 nm using a Cytation5 plate reader. Parasite growth was normalized to untreated control parasites and calculated as a percentage. Background was determined by using uninfected red blood cells.

Dose-dependent assays were performed in at least biological duplicate and technical duplicate. The reported values are averages of the biological replicates with standard error of the mean (SEM). The starting concentrations for the dose-dependent growth inhibition assays were adjusted following the initial screening of the compounds to ensure the EC_50_ was in the middle of the concentration range. The DMSO vehicle was maintained below 0.02% for all tested compounds. The data was fitted using the four-parameter dose-dependent curve and the half-maximal effective concentration (EC_50_) values were calculated using GraphPad Prism (GraphPad Software, Inc.). Assays comparing the EC_50_ values between different cell lines were run in parallel.

### *In Vitro* Selection of Drug Resistance

A single-step strategy was used to select parasites resistant to PRC1590 across three independent cultures as previously reported.^35^ Briefly, a newly cloned 3D7 *P. falciparum* strain was used for the selection of resistance to PRC1590. Three independent inoculums of 1 × 10^7^ parasites in the ring stage (5 mL cultures at 2% parasitemia in 5% hematocrit) were subjected to continuous drug pressure at 1.5 times the EC_50_ value (EC_50_ 3D7 = 145 nM).^25^ A control flask treated with DMSO was set in parallel throughout this selection experiment. For the first 7 days of the selection, media supplemented with PRC1590 was replaced every day. From days 7 to 14, media supplemented with PRC1590 was changed every other day. Drug pressure was removed at day 14 and cultures were monitored by Giemsa-stained smears with media change every other day until recrudescence was observed. Fresh blood (50 μL) was added every seventh day throughout the selection process. After 21 days from the start of drug treatment, parasites emerged in two of the three flasks. Clones from each of the recrudescing flasks were selected by limiting dilution. Resistance to PRC1590 was confirmed with the *in vitro* SYBR Green I assay described above.

### Genomic Analysis

DNA from the five resistant clones and their wild-type parental 3D7 strain were extracted using QIAmp blood mini kit (Qiagen, Germantown, MD). The DNA was concentrated using a DNA clean and concentrator kit from Zymo. Libraries for DNA sequencing were constructed using KAPA HyperPrep Kit (Roche, Indianapolis, Indiana) and sequencing was performed using an Illumina NextSeq2000 PE150 High Output. Due to the A-T richness of the *P. falciparum* genome, 40% PhiX was also added to pooled DNA to improve diversity and sequence quality.^75^ The obtained Illumina read depths were mapped against the *P. falciparum* reference genome assembly, using the BWA mem v.0.7.17 aligner.^76^ The alignment file was then formatted using the package Picard tools to mark duplicate reads for properly submitting the file to the Genome Analysis Toolkit (GATK) v3.8 variant caller.^77^ In this analysis, we set the ploidy equal to one and a minimum Q-score confidence value of 30 for variant calling. The variant call file from GATK was filtered using two steps. The first step used the VariantFiltration tool from GATK to classify SNPs based on their quality. SNP regions with low read depth coverage (<5×), mapping quality <25, and FisherStrand (FS) > 60 were flagged as “hard to validate” regions. The second step applied a strict quality filter considering allele frequency higher than 95% as gold standard SNPs. All SNPs that passed these filters were submitted to a variant annotation and effect prediction via SnpEff v4.3 and vcfannotator to detect affected genes.^78^

### Dose-Dependent Stage Specificity Assays

Stage-specific assays were performed as previously described with some modifications.^44^ Briefly, 3D7 strain parasites were synchronized in the ring stage using three treatments of sorbitol; rings were synchronized twice 48 h before the start of the assay with 6 h between sorbitol treatment. After parasites underwent one lifecycle and reinvaded new red blood cells (RBCs), early rings were again synchronized with sorbitol treatment on the day of the assay. Infected RBCs were then plated in five 96-well plates and sequentially exposed to drug treatment for 8 h starting at early rings (0–8 h), late rings (8–16 h), early trophozoites (16–24 h), late trophozoites (24–32 h), and schizonts (32–40 h). Drug was removed after each exposure through three rounds of washing with RPMI and infected RBCs were transferred to a new plate. Media used for washing was prewarmed to avoid any growth delays. All plates were read 72 h following the start of the assays, and growth inhibition was determined using SYBR Green I assay. Data analysis was performed as described above. Assays were performed in two biological replicates with two technical replicates each.

### Fluo-4 AM Assays

To assess differences in DV import^19^ and DV disruption^52,53^ between the 3D7 strain and the 3D7 PfMDR1 G293V line parasites at 20–24 h postinfection were stained with Fluo-4 AM (Thermo Fisher Scientific, Waltham, MA) as previously described with some modifications.^19,51,52^ Briefly, parasites were synchronized in the ring stage by two rounds of sorbitol treatment. Once in the trophozoite stage, 2.5 mL cultures at 5% hematocrit and a parasitemia greater than 5% were washed and resuspended in 2.5 mL of Ringer’s solution (122.5 mM NaCl, 5.4 mM KCl, 1.2 mM CaCl_2_, 0.8 mM MgCl_2_, 11 mM D-glucose, 10 mM HEPES, 1 mM NaH_2_PO_4_, pH 7.4; all reagents from Sigma-Aldrich). Cultures were then transferred to a flask and incubated with 1 μM Fluo-4 AM for 30 min at 37 °C in reduced oxygen conditions, protected from light. Following incubation, cultures were washed twice and resuspended in Ringer’s solution. Images were obtained immediately within 1 h after the final wash. To assess if PRC1590 treatment affected the Fluo-4 intensity in the DV, 3D7 wild-type and 3D7 PfMDR1 G293V parasites were treated with 1 μM PRC1590 or an equivalent volume of DMSO in RPMI for 4 h in a 12.5 cm^2^ flask (5% hematocrit with a parasitemia greater than 5%), shaking at 37 °C under reduced oxygen conditions. Chloroquine treatment at 1 μM for 4 h was used as control. Following drug treatment, cultures were washed three times with RPMI followed by resuspension in Ringer’s solution containing 1 μM Fluo-4 AM and incubated for 30 min at 37 °C shaking under reduced oxygen conditions and protected from light. Images were obtained immediately within 1 h after the final wash.

For imaging, parasites were placed in 35 mm glass dishes coated with poly-l-lysine, and images were obtained using a Zeiss LSM 980 Confocal Microscope. Z-Stack images were obtained by using a 100x objective. Confocal microscopy settings were optimized to include a 488 nm diode laser for capturing Fluo-4 fluorescence at 2% transmission and a detector gain of 900 V, and brightfield (PMT detector) at a detector gain of 350 V. Thirty images per condition from at least three biological replicates were obtained for the 3D7 strain and the 3D7 PfMDR1 G293V line with no drug treatment and treatment with DMSO, PRC1590, or chloroquine. Zen Blue software was used to adjust the brightness and contrast uniformly across images and conditions for display purposes. To quantify fluorescence intensity, the measure function in ImageJ was used.^79^ To determine the mean fluorescence intensity, intensity was measured three times for each image. ANOVA and Šídák’s multiple comparisons test were run to statistically compare the fluorescence intensities between the parasite cell lines and treatment conditions using GraphPad Prism.

## Supporting Information Available

Resistance phenotype stability (Figure S1); growth phenotype assessments of 3D7, 3D7^1590R1^, and 3D7^1590R4^ (Figure S2); resistome library of selected compounds from the Malaria Box (Table S1); EC_50_ values of the MMV compounds identified in the cross-resistance screening and common antimalarials (Table S2); amino acid sequence alignment for PfMDR1 from 3D7^WT^ and 3D7^1590R1–5^ lines (Figure S3); stage specificity assays for *P. falciparum* treated with chloroquine and morphological assessments of 3D7 strain treated with chloroquine and PRC1590 (Figure S4); additional images of the Fluo-4 assays (Figures S5–S11); additional materials and methods; and all genomic variants identified in each of the individual lines compared to the 3D7 reference strain in PlasmoDB as well as the identified genomic variants in the resistant clones with respect to 3D7-WT (Table S3) (PDF)
All genomic variants, selected genome variants (XLSX)
