# Supplementary material for: Stereospecific Resistance to N2-Acyl Tetrahydro-β-carboline Antimalarials Is Mediated by a PfMDR1 Mutation That Confers Collateral Drug Sensitivity
Source: ACS Infect Dis. 2025 Jan 14;11(2):529–42. doi: 10.1021/acsinfecdis.4c01001 (PMC11828674; doi:10.1021/acsinfecdis.4c01001)
Supplement: Supplementary file 1 — id4c01001_si_001.pdf [file id4c01001_si_001.pdf]

Supporting Information

Stereospecific resistance to N2-acyl tetrahydro-β-carboline antimalarials is mediated by a PfMDR1 mutation that confers collateral drug sensitivity

Emily K. Bremers[1,2], Joshua H. Butler[1,2], Leticia S. Do Amaral[1,2], Emilio F. Merino[1,2], Hanan Almolhim[3], Bo Zhou[4], Rodrigo P. Baptista[5], Maxim Totrov[6], Paul R. Carlier[3,4], and Maria Belen Cassera[1,2]*

[1]Department of Biochemistry and Molecular Biology, University of Georgia, Athens, GA 30602
[2]Center for Tropical and Emerging Global Diseases, University of Georgia, Athens, GA 30602
[3]Department of Chemistry, Virginia Tech, Blacksburg, VA 24061
[4]Department of Pharmaceutical Sciences, University of Illinois Chicago, Chicago, IL 60612
[5]Department of Medicine, Houston Methodist Research Institute, Houston, TX 77030
[6]MolSoft LLC, San Diego, California 92121
*Correspondence: maria.cassera@uga.edu

Table of Contents

Figure S1	Page S2
Figure S2	Page S3
Table S1	Page S4
Table S2	Page S5
Figure S3	Page S6
Figure S4	Page S7
Figure S5	Page S8
Figure S6	Page S9
Figure S7	Page S10
Figure S8	Page S11
Figure S9	Page S12
Figure S10	Page S13
Figure S11	Page S14
Materials and Methods	Page S15
References	Page S17

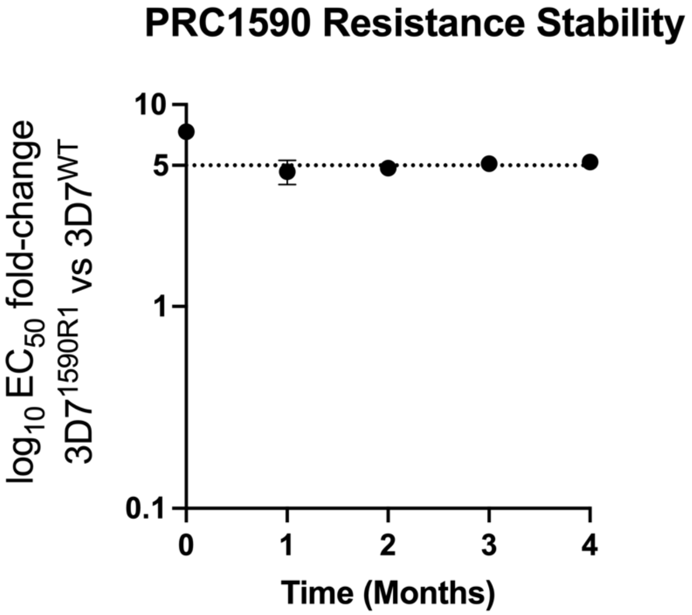

Month	$3D7^{1590R1}$ EC_{50} ± S.D. [nM]	$3D7^{WT}$ EC_{50} ± S.D. [nM]
0	1279 ± 82	176
1	524 ± 119	112 ± 12
2	872 ± 29	179 ± 24
3	986 ± 102	198 ± 1
4	1475 ± 35	283 ± 8

Figure S1. The stability of the resistance phenotype was monitored for 4 months in the absence of drug pressure after PRC1590 resistant line was selected. Initially, $3D7^{1590R1}$ had a fold-change of 7 x EC_{50} value compared to $3D7^{WT}$ and stabilized at 5 x EC_{50} value in subsequent months. The y-axis is shown in log scale. The results represent averages and S.D. of three technical replicates.

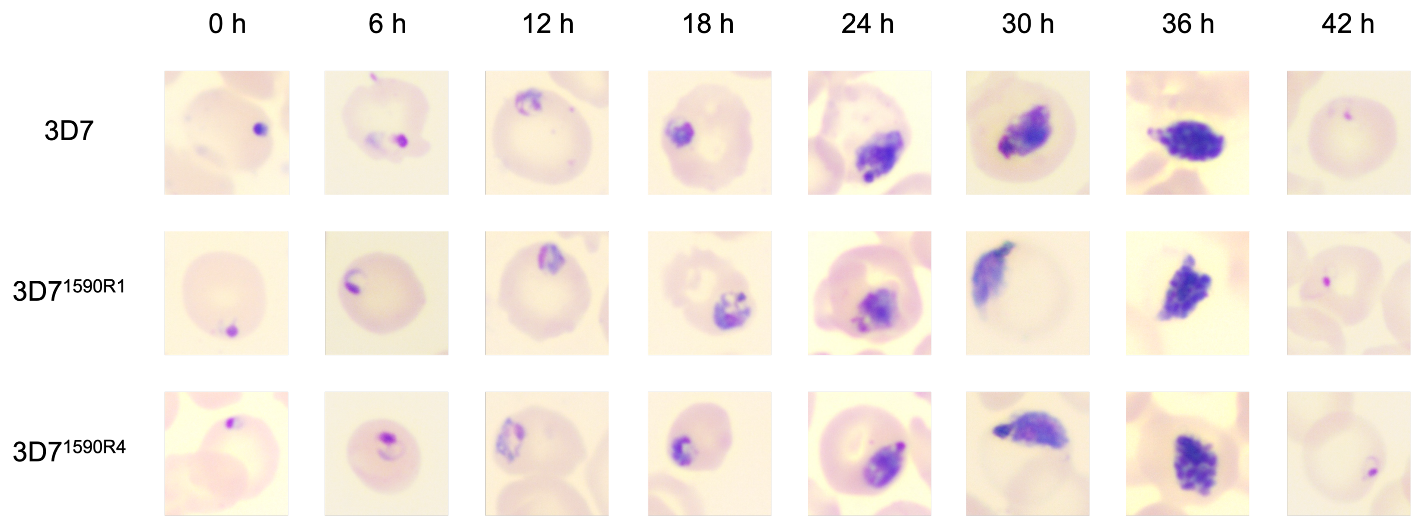

Figure S2. Growth phenotypes were assessed for the resistant strains $3D7^{1590R1}$ and $3D7^{1590R4}$ in comparison to $3D7^{WT}$ (wild-type). Parasites were tightly synchronized with three cycles of sorbitol and blood smears were taken every 6 hours throughout the intraerythrocytic lifecycle. There was no discernable difference in growth phenotype between the PRC1590 resistant lines ($3D7^{1590R1}$ and $3D7^{1590R4}$) and the parental strain ($3D7^{WT}$).

Table S1. Resistome library of selected compounds from the Malaria Box used to identify common mechanisms of resistance and their associated genes.

MMV compound ID	Known gene(s) to mediate resistance (PlasmoDB Gene ID) [Citation]
MMV009108	*pflss* (PF3D7_0107500) [1]<br>*pfatp4* (PF3D7_1211900) [2]
MMV007564	*pfcarl* (PF3D7_0321900) [1]
MMV665939	*pfap2tf-6b* (PF3D7_0613800) [1]<br>*pfabci3* (PF3D7_0319700) [1]
MMV019066	*pfftb* (PF3D7_1147500) [1]
MMV006767	*pfmate* (PF3D7_0212800) [1]<br>*pfcrt* (PF3D7_0709000) [1]
MMV011895	*pfaat1* (PF3D7_0629500) [1]<br>*pfcrt* (PF3D7_0709000) [1]
MMV665882	*pfmdr1 (*PF3D7_0523000) [1]<br>*pfap2tf-6b* (PF3D7_0613800) [1]
MMV007224	*pfatpase2* (PF3D7_1219600) [1]<br>*pfaat2* (PF3D7_1208400) [1]<br>*pfsamc* (PF3D7_1241600) [1]<br>*pfcaf16 (*PF3D7_1434000) [1]<br>*pfaat1* (PF3D7_0629500) [1]
MMV665924	*pfacs11* (PF3D7_1238800) [1]<br>*pfabcb4* (PF3D7_0302600) [1]<br>*pfacs10* (PF3D7_0525100) [1]
MMV019313	bifunctional farnesyl/geranylgeranyl diphosphate synthase (PF3D7_1128400) [3]
MMV009063	*pfmdr1* (PF3D7_0523000) [1]<br>*pfpi4k* (PF3D7_0509800) [1]
MMV019017	*pfmdr1* (PF3D7_0523000) [1]<br>*pfpi4k* (PF3D7_0509800) [1]
MMV665789	*pfmdr1* (PF3D7_0523000) [1]<br>*pfpi4k* (PF3D7_0509800) [1]<br>*pfmdr2* (PF3D7_1447900) [1]
MMV019662	*pflss* (PF3D7_0107500) [1]<br>*pfmcp1* (PF3D7_0108400) [1]
MMV008149	*pfap2tf-10* (PF3D7_1007700) [1]<br>*pfcytb* (mal_mito_3) (PfNF54_000013000) [1]<br>*pfmrp1* (PF3D7_0112200) [1]
MMV024114	*pfabci3* (PF3D7_0319700) [1]<br>*pfcrt* (PF3D7_0709000) [1]
MMV011438	*pfap2tf-10* (PF3D7_1007700) [1]<br>*pfpare* (PF3D7_0709700) [1]
MMV665852	*pfatpase2* (PF3D7_1219600) [1]

Table S2. EC_{50} values for the MMV compounds identified in the cross-resistance screening and common antimalarials. Assays for all three strains were conducted in parallel. Due to limited supply of MMV compounds, only one PRC1590 resistant line was assessed ($3D7^{1590R1}$) and assays with MMV compounds represent data from two biological replicates conducted in technical duplicate. All other data represent at least three biological replicates conducted in technical duplicate. n.d. indicates “not determined”.

Compound	$3D7^{1590R1}$ EC_{50} ± S.E.M. [nM]	$3D7^{1590R4}$ EC_{50} ± S.E.M. [nM]	$3D7^{WT}$ EC_{50} ± S.E.M. [nM]	$Dd2^{WT}$ EC_{50} ± S.E.M. [nM]
PRC1590	770 ± 50	797 ± 5	145 ± 10	142 ± 21
MMV009063	118 ± 19	n.d.	400 ± 4	363 ± 36
MMV019017	187 ± 9	n.d.	554 ± 6	773 ± 81
MMV665789	287 ± 26	n.d.	889 ± 98	934 ± 287
MMV665882	46 ± 3	n.d.	74 ± 2	165 ± 7
MMV665852	1634 ± 48	n.d.	1164 ± 23	1299 ± 158
Chloroquine	14 ± 2	11 ± 2	13 ± 2	251 ± 52
Quinine	22 ± 1	30 ± 3	58 ± 8	565 ± 137
Quinidine	7 ± 1	7 ± 1	16 ± 1	9 ± 1
Mefloquine	7 ± 1	7 ± 2	17 ± 3	17 ± 2
Amodiaquine	7 ± 1	9 ± 1	8 ± 1	8 ± 1

Figure S3. Amino acid sequence alignment for PfMDR1 from Pf3D7_0709700 (PlasmoDB), 3D7^{WT} and 3D7$^{1590R1-5}$ lines. The single amino acid change on the 293rd residue of the PfMDR1 protein (G293V) in 3D7$^{1590R1-5}$ lines is indicated in red.

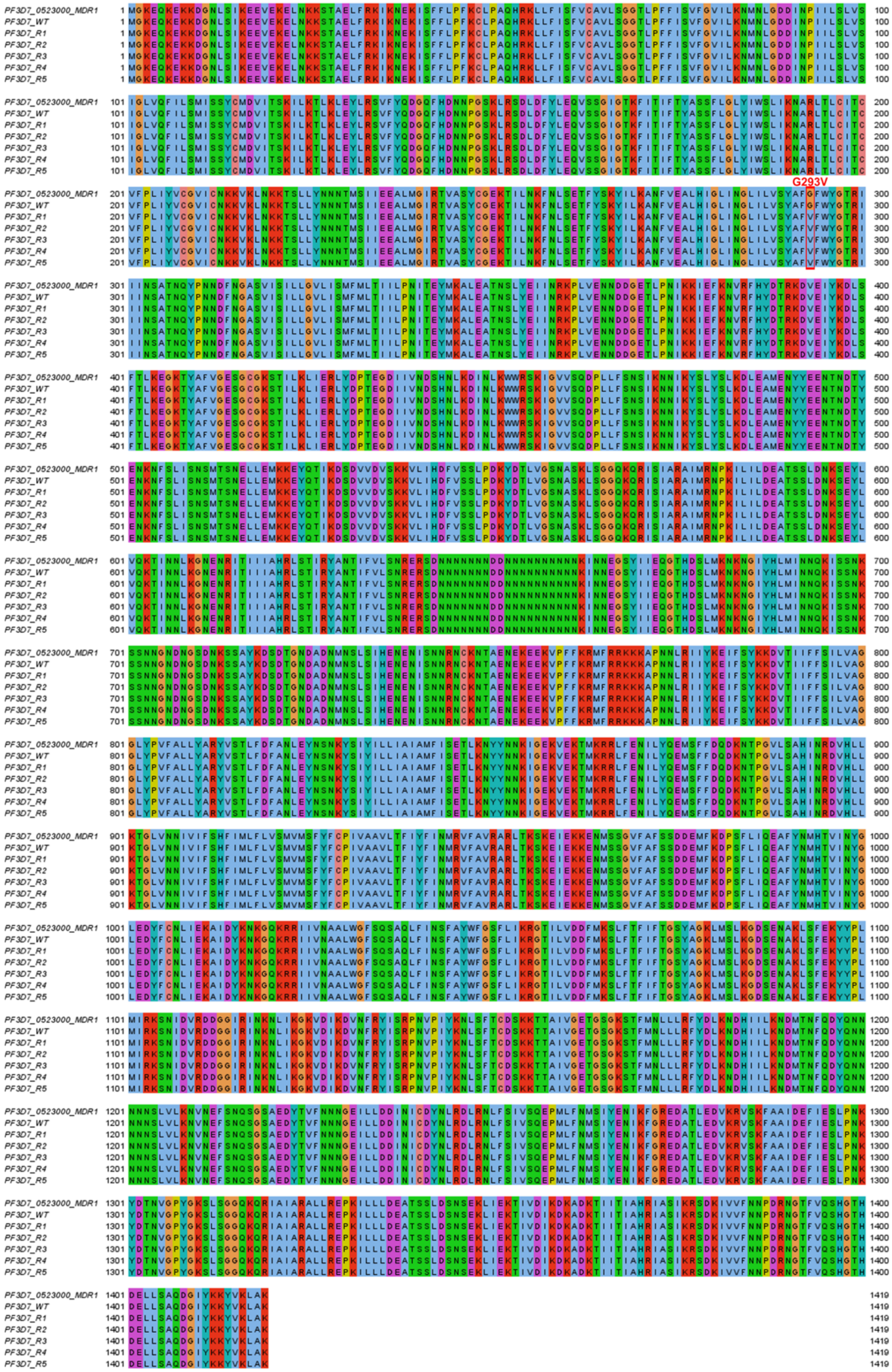

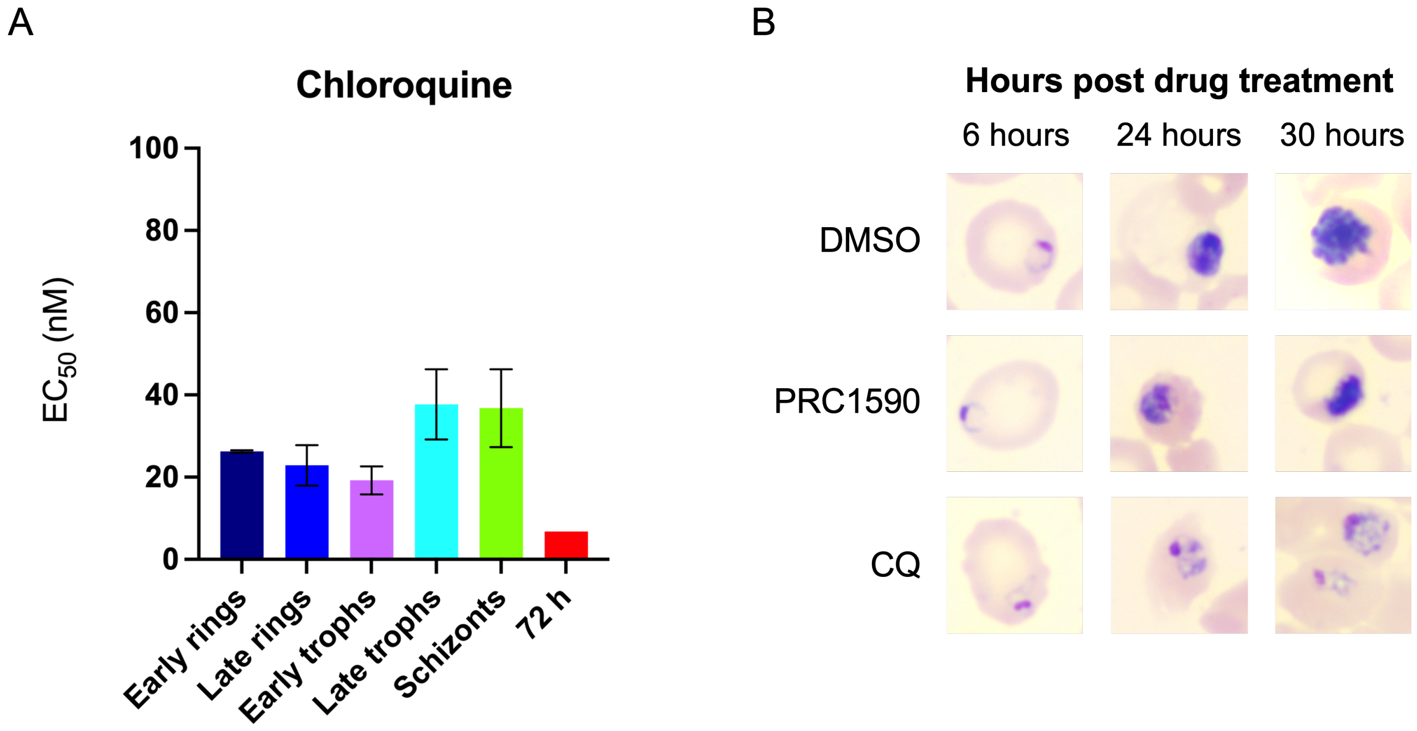

Figure S4. Comparison of Chloroquine and PRC1590. **A)** Stage specificity assay for chloroquine showing that this compound acts most potently in the late ring and early trophozoite stages, as previously shown by Murithi and colleagues [4]. The EC_{50} values represent data from two biological replicates conducted in technical duplicate. **B)** Tightly synchronized ring stage 3D7 strain parasites were treated with PRC1590 and chloroquine (CQ) at 5 x EC_{50} value. Parasites treated with 0.05% DMSO was run in parallel as a control. PRC1590 treatment results in trophozoites with a condensed morphology, while treatment with CQ results in an enlarged digestive vacuole.

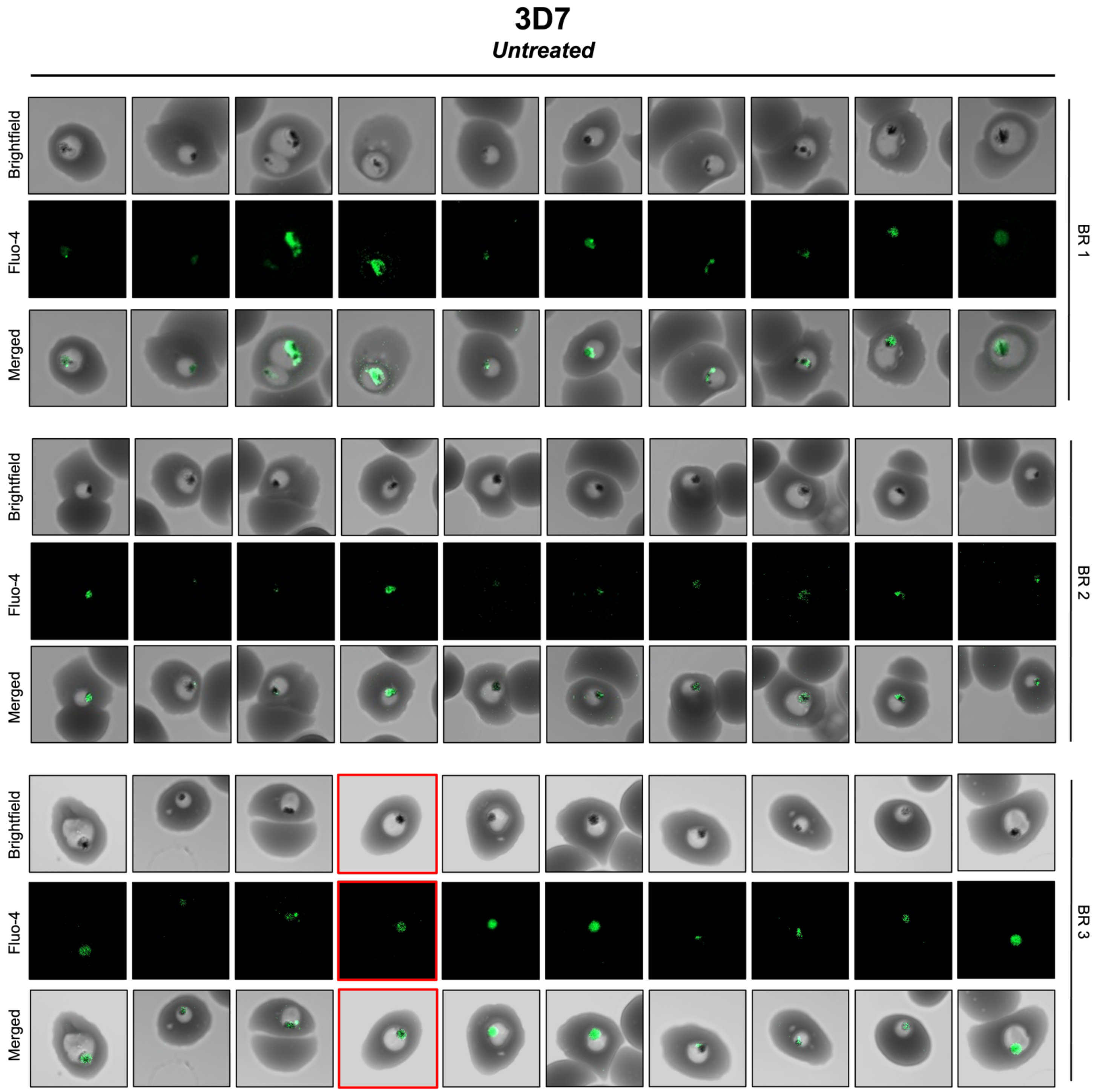

Figure S5. Fluo-4 import assays performed in the 3D7 strain. Fluo-4 fluoresces when bound to calcium and is mainly detected in the digestive vacuole (DV) of the malaria parasite. As previously described, the 3D7 strain of *P. falciparum* shows strong Fluo-4 accumulation in the DV of the parasite, with low localization outside this organelle [5]. Data represents n = 30 images across three biological replicates (BR 1-3). The red border indicates images shown in Figure 6.

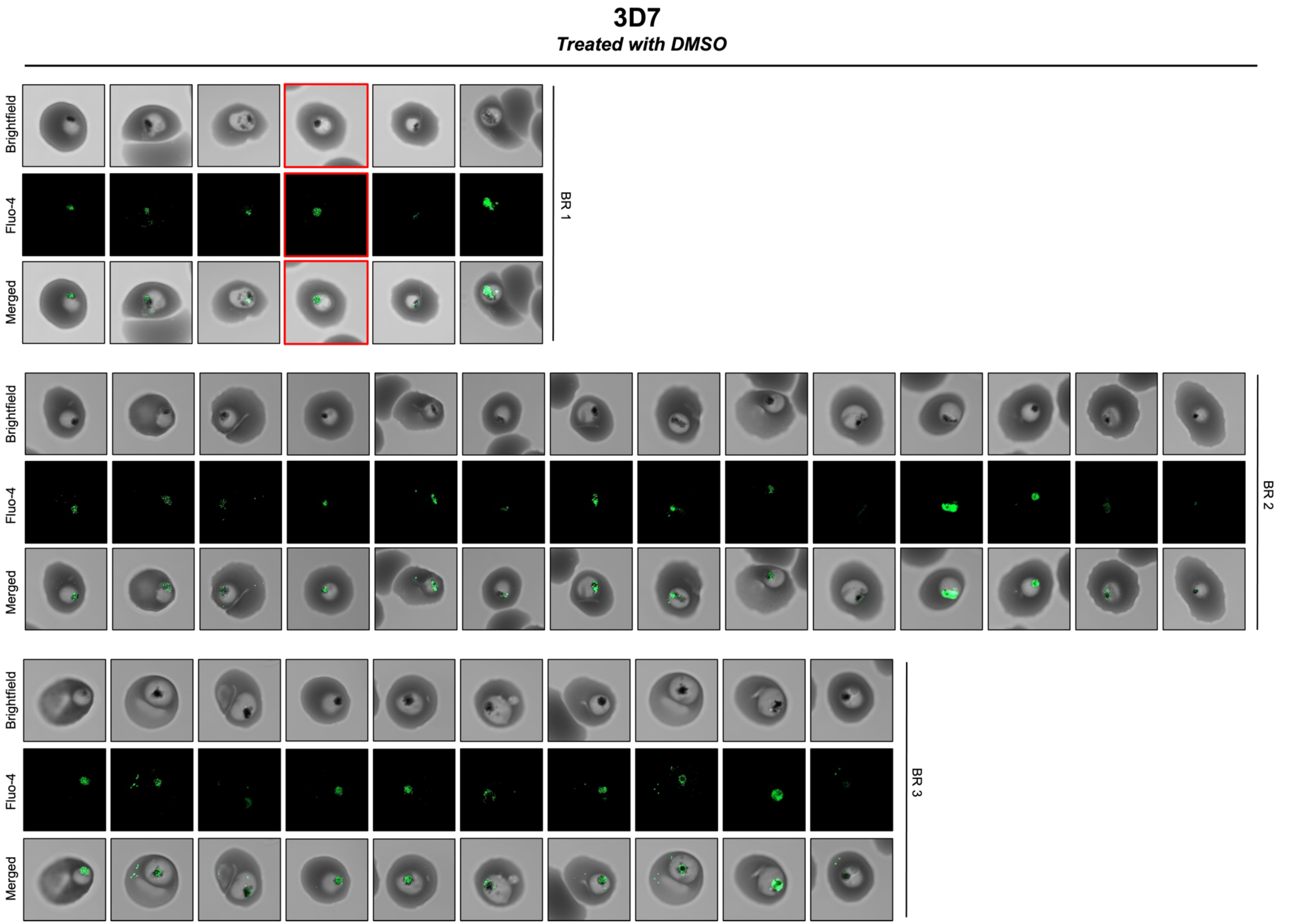

Figure S6. Fluo-4 localization in the 3D7 strain when treated with DMSO used as a negative control for treatment with PRC1590. Data represents n = 30 images across three biological replicates (BR 1-3). The red border indicates images shown in Figure 6.

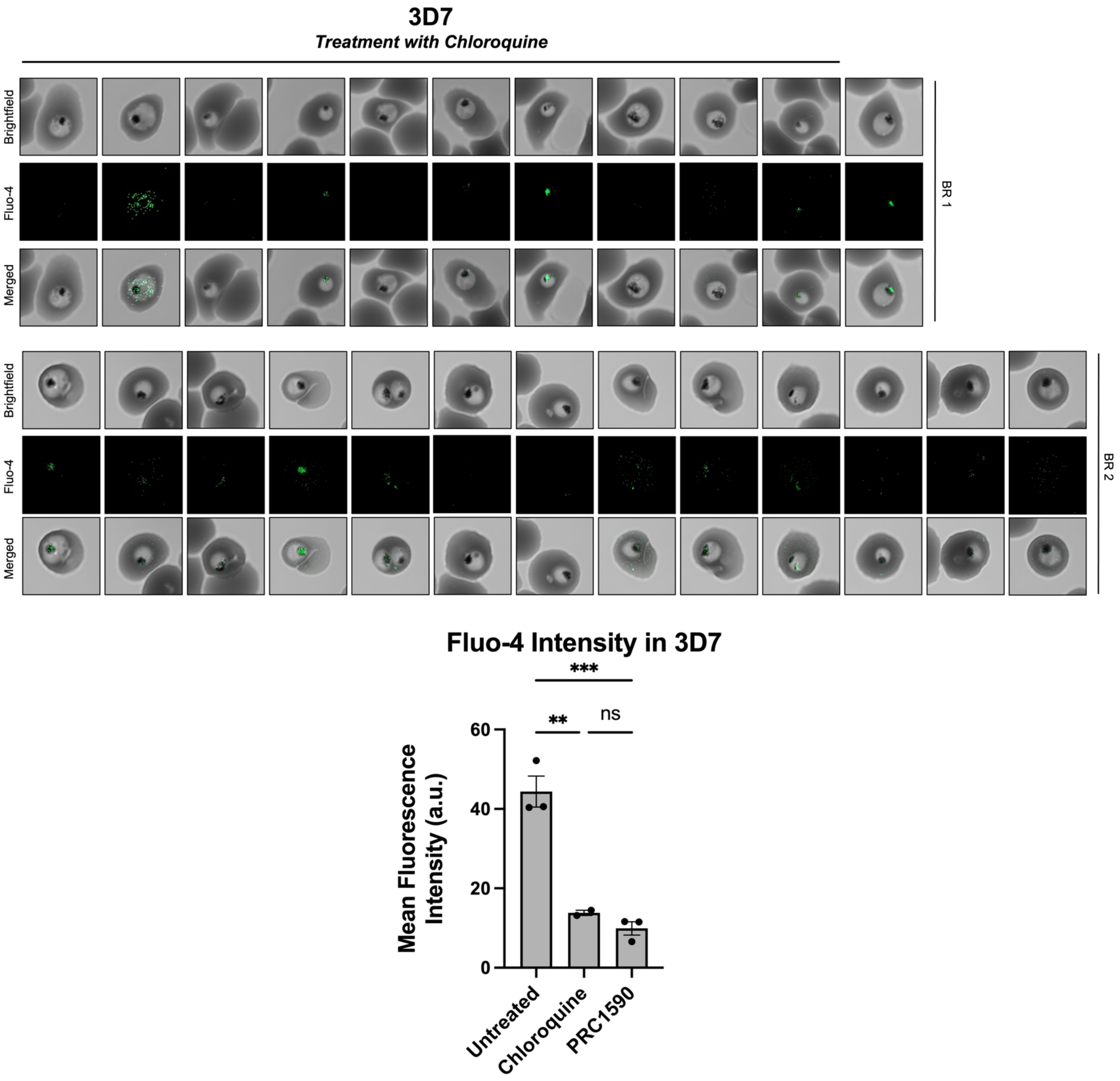

Figure S7. DV disruption assays using Fluo-4 AM in the 3D7 wild-type strain treated with treated with 1 μM of chloroquine. These experiments were used as a positive control for assessing DV calcium disruption with Fluo-4. Data represents n = 24 images across two biological replicates (BR 1-2). Bar graph comparing the mean ± S.E.M values of fluorescence intensity in the 3D7 untreated control (no drug), chloroquine and PRC1590 treated parasites. Treatment with chloroquine significantly reduced Fluo-4 intensity when compared to the untreated control. No significant difference was observed between chloroquine and PRC1590 treatments. A one-way ANOVA test was performed to compare treatment conditions in 3D7. ****p=0.0006, ***p=0.0019, ns indicates not significant.

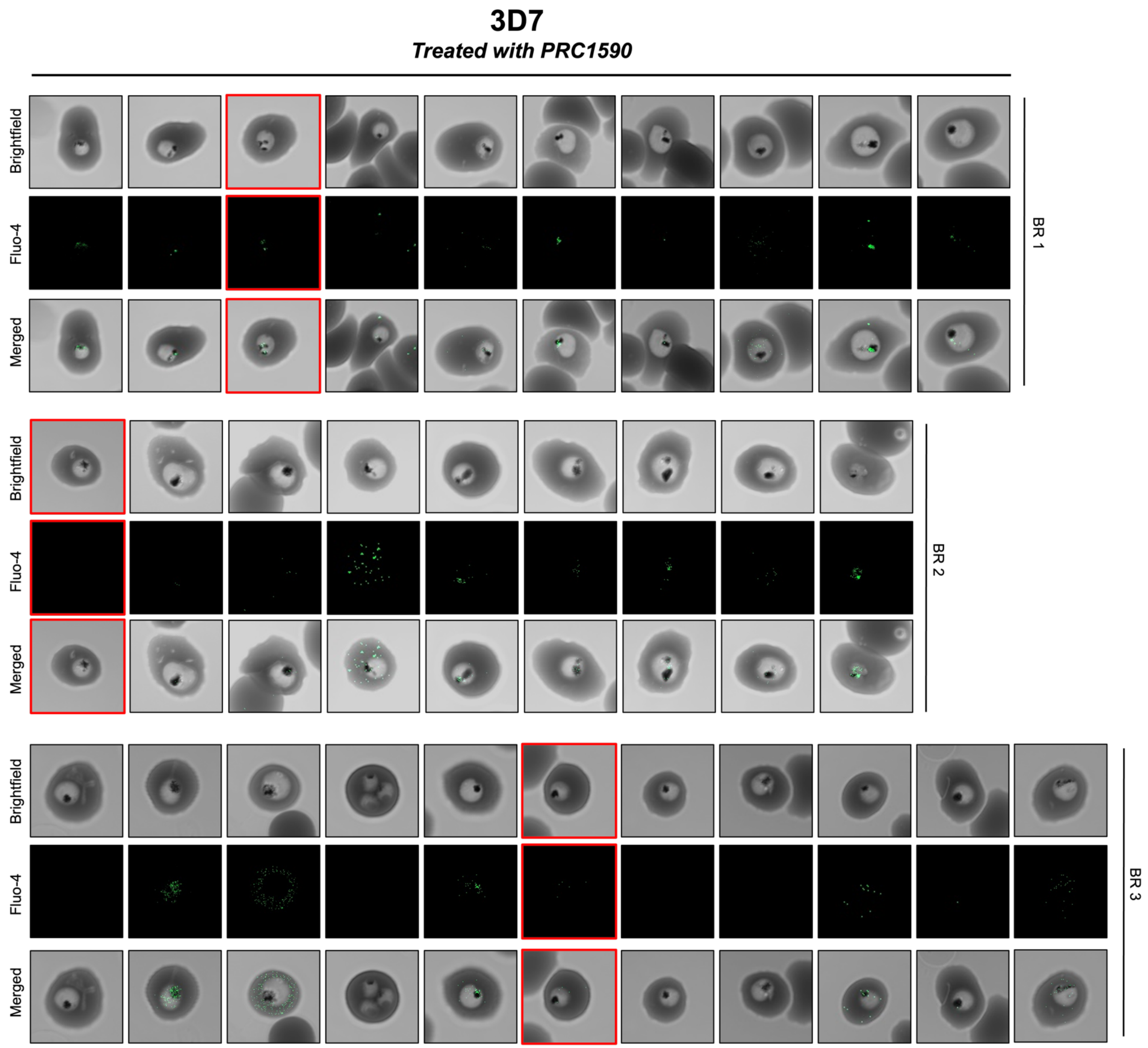

Figure S8. DV disruption assays performed in the 3D7 strain treated with 1 μM PRC1590. Data represents n = 30 images across three biological replicates (BR 1-3). The red border indicates images shown in Figure 6.

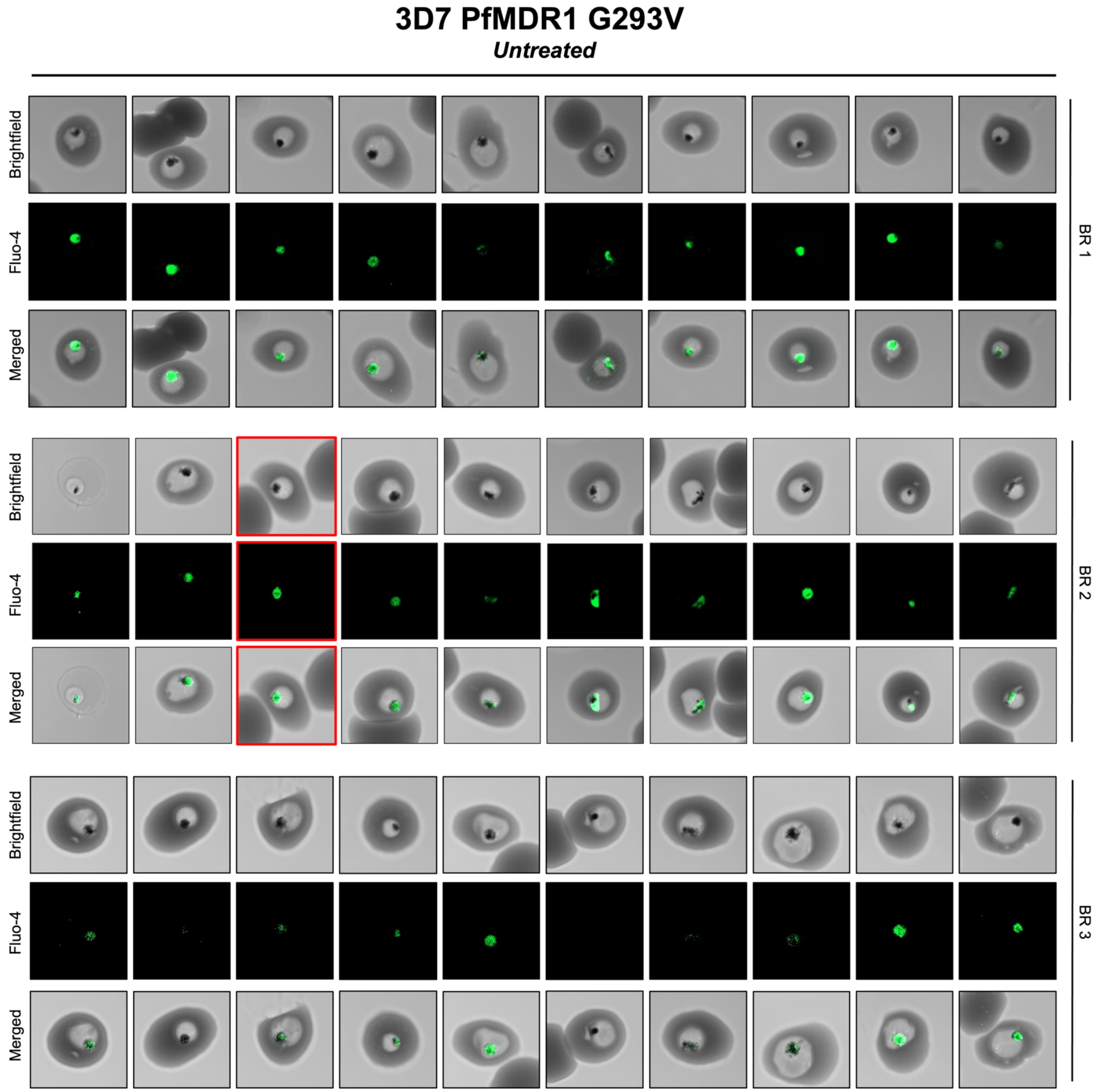

Figure S9. Fluo-4 import assays performed in the 3D7 PfMDR1 G293V line. These data suggest that the PfMDR1 G293V mutation does not impact import of Fluo-4 into the DV when compared to the untreated control or DMSO treated condition. Data represents n = 30 images across three biological replicates (BR 1-3). The red border indicates images shown in Figure 6.

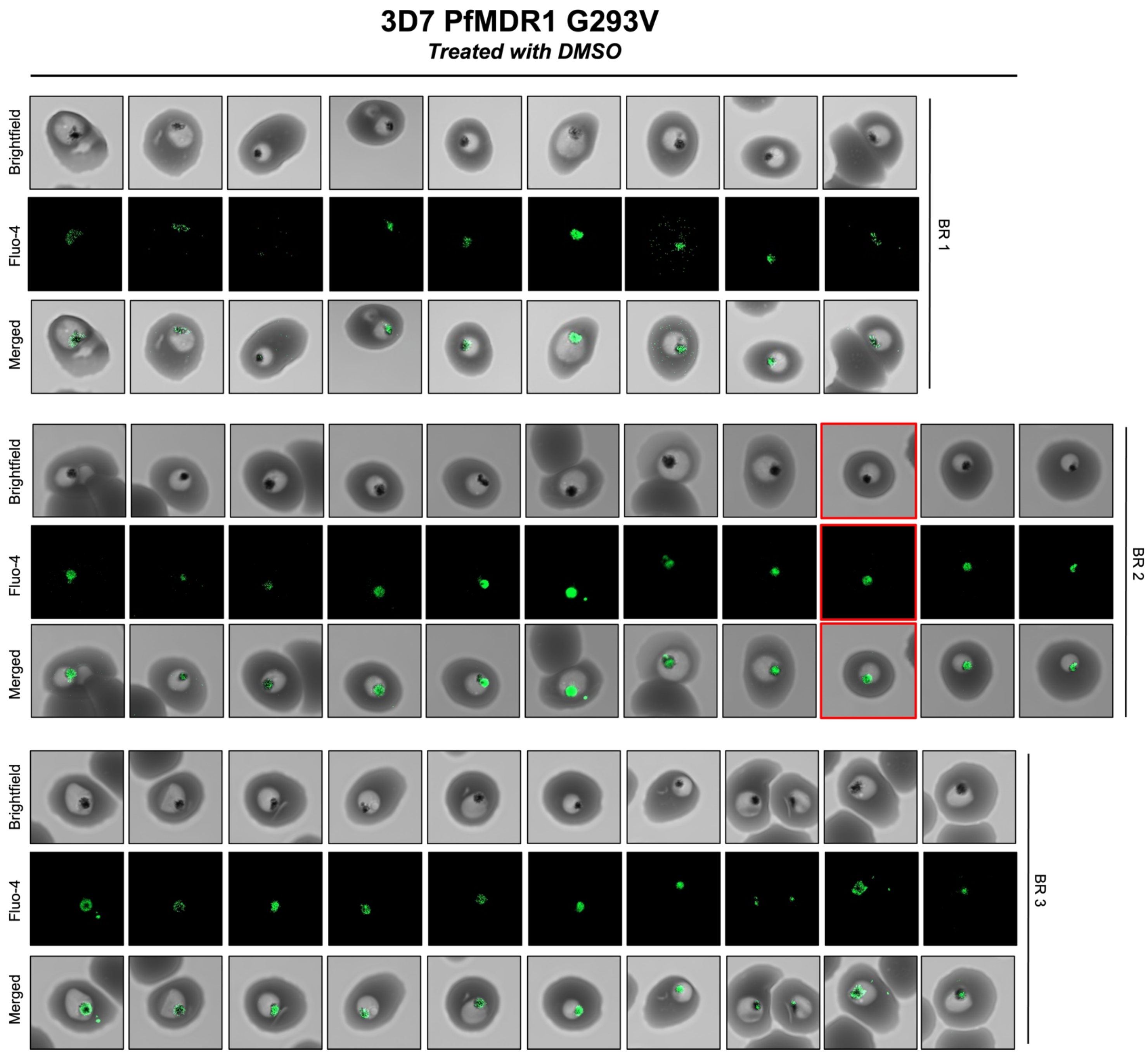

Figure S10. DV disruption assays performed in the 3D7 PfMDR1 G293V line treated with DMSO used as a negative control. Data represents n = 30 images across three biological replicates (BR 1-3). The red border indicates images shown in Figure 6.

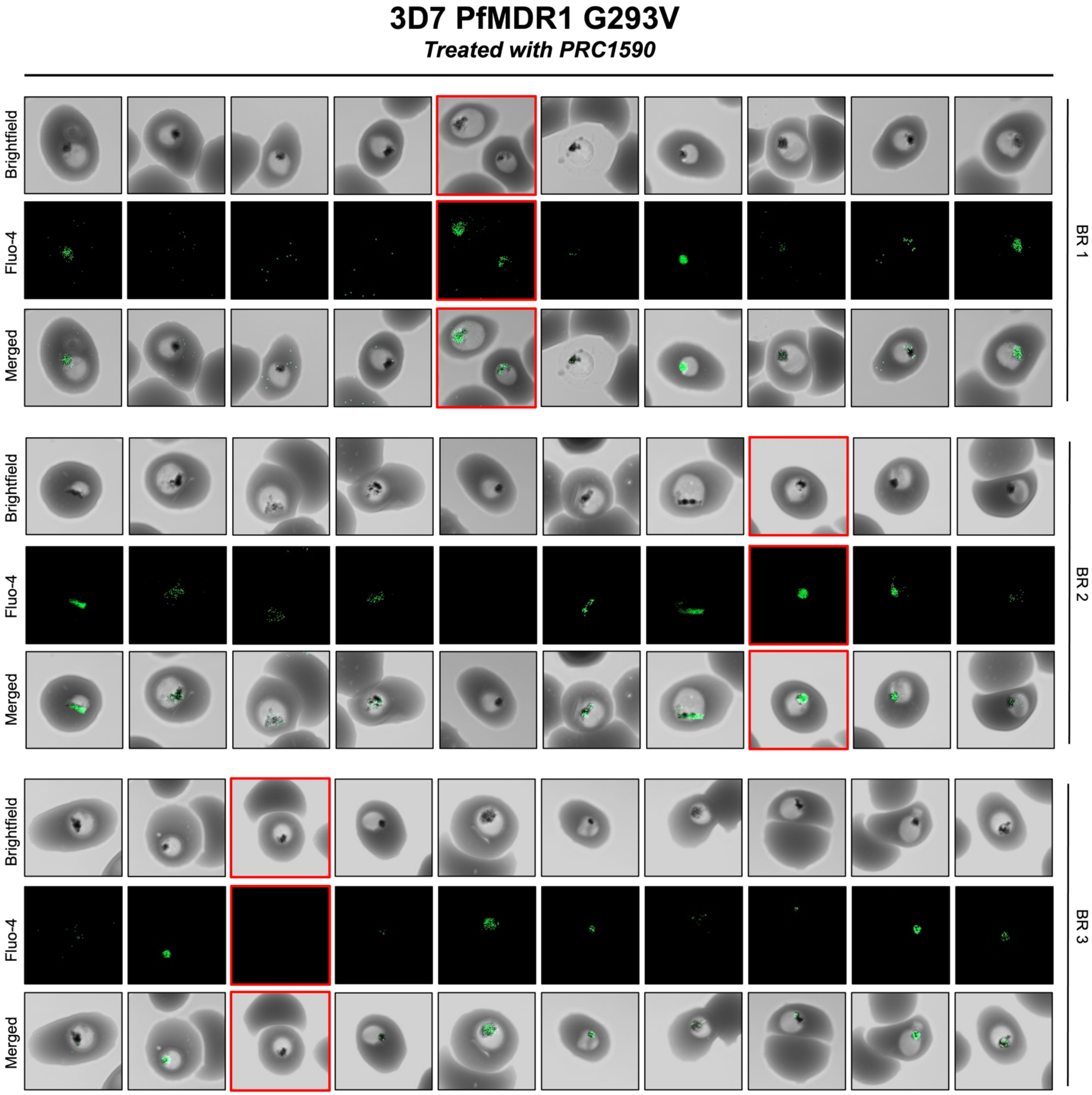

Figure S11. DV disruption assays performed in the 3D7 PfMDR1 G293V line treated with 1 μM PRC1590. These data show a slight reduction in Fluo-4 fluorescence intensity compared to the DMSO control, suggesting that DV disruption is also occurring in this resistant cell line. In comparison to its parental line, 3D7 PfMDR1 G293V is better able to tolerate PRC1590 treatment potentially by mitigating the disruption of the DV structure or function. Data represents n = 30 images across three biological replicates (BR 1-3). The red border indicates images shown in Figure 6.

Materials and Methods

Chemicals

The Malaria Box compounds were obtained through Medicines for Malaria Venture (MMV, Switzerland) and were supplied at 10 mM in DMSO. Quinine, chloroquine, mefloquine were obtained from Sigma-Aldrich (St. Louis, MO, USA), and amodiaquine was obtained from Cayman Chemical Company (Ann Arbor, MI, USA). All compounds were reconstituted in DMSO, except for chloroquine which was reconstituted in water. PRC1590, PRC1589, PRC1617 and PRC1618 were synthesized and purified, as previously described[6].

Plasmodium falciparum cultures

The *P. falciparum* strains 3D7 (MRA-102) and Dd2 (MRA-150) were obtained from MR4 (ATCC, Manassas, VA, USA; BEI Resources, NIAID, NIH). The *P. falciparum* Dd2 PfMDR1 A807V line was kindly supplied by Caroline Ng (University of Nebraska Medical Center, USA)[7]. Cultures were maintained at 5% hematocrit in O positive blood (Grifols, Memphis, TN, USA) and RPMI 1640 media (Thermo Fisher Scientific, Waltham, MA, USA) containing 2 g/L glucose, 5.94 g/L HEPES, 2.3 g/L sodium bicarbonate, 5 g/L Albumax I, and 50 mg/L hypoxanthine, all obtained from Sigma-Aldrich (St. Louis, MO, USA). Media was supplemented with 20 mg/L of gentamicin (Thermo Fisher Scientific, Waltham, MA, USA). Parasite cultures were kept at 37 °C, shaking, in reduced oxygen conditions (5% CO_2, 5% O_2, and 90% N_2). Synchronous ring stage cultures were obtained through two rounds of treatment with 5% sorbitol (Sigma-Aldrich, St. Louis, MO, USA), performed 6 h apart.

Plasmodium falciparum Morphological Assessments

Two resistant clones, $3D7^{1590R1}$ and $3D7^{1590R4}$ derived from two independent selections, were used to assess potential growth phenotype differences compared to their parental line ($3D7^{WT}$). Tightly synchronized ring stage parasite cultures were obtained by three sequential treatments of 5% sorbitol (Sigma-Aldrich, St. Louis, MO, USA) prior to the start of the experiment (-48, -40, and 0 hours). All cultures were then set at 3% parasitemia, 5% hematocrit, and kept at 37 °C with shaking in reduced oxygen conditions (5% CO_2, 5% O_2, and 90% N_2). Thin blood smears were performed every 6 hours, fixed with 100% methanol (Sigma-Aldrich, St. Louis, MO, USA) and stained for 15 min in 20% Giemsa (Sigma-Aldrich; diluted in deionized water). Morphological growth phenotypes were performed in biological duplicate.

To assess morphological changes in parasites under drug treatment, we subjected 3D7 strain parasites at 3% parasitemia and 5% hematocrit to treatment with PRC1590 and chloroquine. Parasites were tightly synchronized in the ring stage with three rounds of 5% sorbitol before being subjected to 5 times the EC_{50} values of PRC1590 and chloroquine. A control flask treated with an equal volume of DMSO was used as a control. Smears were taken throughout the intraerythrocytic lifecycle to assess potential morphological differences between drug treatments. Following performing thin blood smears, parasites were immediately gassed and returned to 37 °C with shaking. Morphological phenotype assessments under drug treatment were performed in biological triplicate.

References

(1) Cowell, A. N.; Istvan, E. S.; Lukens, A. K.; Gomez-Lorenzo, M. G.; Vanaerschot, M.; Sakata-Kato, T.; Flannery, E. L.; Magistrado, P.; Owen, E.; Abraham, M.; LaMonte, G.; Painter, H. J.; Williams, R. M.; Franco, V.; Linares, M.; Arriaga, I.; Bopp, S.; Corey, V. C.; Gnädig, N. F.; Coburn-Flynn, O.; Reimer, C.; Gupta, P.; Murithi, J. M.; Moura, P. A.; Fuchs, O.; Sasaki, E.; Kim, S. W.; Teng, C. H.; Wang, L. T.; Akidil, A.; Adjalley, S.; Willis, P. A.; Siegel, D.; Tanaseichuk, O.; Zhong, Y.; Zhou, Y.; Llinás, M.; Ottilie, S.; Gamo, F.-J.; Lee, M. C. S.; Goldberg, D. E.; Fidock, D. A.; Wirth, D. F.; Winzeler, E. A. Mapping the Malaria Parasite Druggable Genome by Using in Vitro Evolution and Chemogenomics. *Science* **2018**, *359* (6372), 191–199. https://doi.org/10.1126/science.aan4472.

(2) Corey, V. C.; Lukens, A. K.; Istvan, E. S.; Lee, M. C. S.; Franco, V.; Magistrado, P.; Coburn-Flynn, O.; Sakata-Kato, T.; Fuchs, O.; Gnädig, N. F.; Goldgof, G.; Linares, M.; Gomez-Lorenzo, M. G.; De Cózar, C.; Lafuente-Monasterio, M. J.; Prats, S.; Meister, S.; Tanaseichuk, O.; Wree, M.; Zhou, Y.; Willis, P. A.; Gamo, F.-J.; Goldberg, D. E.; Fidock, D. A.; Wirth, D. F.; Winzeler, E. A. A Broad Analysis of Resistance Development in the Malaria Parasite. *Nat Commun* **2016**, *7* (1), 11901. https://doi.org/10.1038/ncomms11901.

(3) Gisselberg, J. E.; Herrera, Z.; Orchard, L. M.; Llinás, M.; Yeh, E. Specific Inhibition of the Bifunctional Farnesyl/Geranylgeranyl Diphosphate Synthase in Malaria Parasites via a New Small-Molecule Binding Site. *Cell Chemical Biology* **2018**, *25* (2), 185-193.e5. https://doi.org/10.1016/j.chembiol.2017.11.010.

(4) Murithi, J. M.; Owen, E. S.; Istvan, E. S.; Lee, M. C. S.; Ottilie, S.; Chibale, K.; Goldberg, D. E.; Winzeler, E. A.; Llinás, M.; Fidock, D. A.; Vanaerschot, M. Combining Stage Specificity and Metabolomic Profiling to Advance Antimalarial Drug Discovery. *Cell Chemical Biology* **2020**, *27* (2), 158-171.e3. https://doi.org/10.1016/j.chembiol.2019.11.009.

(5) Reiling, S. J.; Rohrbach, P. Monitoring PfMDR1 Transport in Plasmodium Falciparum. *Malar J* **2015**, *14* (1), 270. https://doi.org/10.1186/s12936-015-0791-3.

(6) Almolhim, H.; Ding, S.; Butler, J. H.; Bremers, E. K.; Butschek, G. J.; Slebodnick, C.; Merino, E. F.; Rizopoulos, Z.; Totrov, M.; Cassera, M. B.; Carlier, P. R. Enantiopure Benzofuran-2-Carboxamides of 1-Aryltetrahydro-β-Carbolines Are Potent Antimalarials *In Vitro*. *ACS Med. Chem. Lett.* **2022**, acsmedchemlett.1c00697. https://doi.org/10.1021/acsmedchemlett.1c00697.

(7) Ng, C. L.; Siciliano, G.; Lee, M. C. S.; de Almeida, M. J.; Corey, V. C.; Bopp, S. E.; Bertuccini, L.; Wittlin, S.; Kasdin, R. G.; Le Bihan, A.; Clozel, M.; Winzeler, E. A.; Alano, P.; Fidock, D. A. CRISPR-Cas9-Modified *Pfmdr1* Protects *Plasmodium Falciparum* Asexual Blood Stages and Gametocytes against a Class of Piperazine-Containing Compounds but Potentiates Artemisinin-Based Combination Therapy Partner Drugs: Targeting PfMDR1 to Treat Resistant *P. Falciparum*. *Molecular Microbiology* **2016**, *101* (3), 381–393. https://doi.org/10.1111/mmi.13397.
